# Signals of vagal circuits engaging with AKT1 in α7 nAChR^+^CD11b^+^ cells lessen *E. coli* and LPS-induced acute inflammatory injury

**DOI:** 10.1038/celldisc.2017.9

**Published:** 2017-04-11

**Authors:** Caiqi Zhao, Xi Yang, Emily M Su, Yuanyuan Huang, Ling Li, Michael A Matthay, Xiao Su

**Affiliations:** 1 CAS Key Laboratory of Molecular Virology & Immunology, Unit of Respiratory Infection and Immunity, Institut Pasteur of Shanghai, Chinese Academy of Sciences, University of Chinese Academy of Sciences, Shanghai, China; 2 Dana Farber Cancer Institute, Boston, MA, USA; 3 Cardiovascular Research Institute, University of California, San Francisco, CA, USA

**Keywords:** Vagal circuits, Î±7 nicotinic acetylcholine receptor, AKT1, CD11b, acute lung injury

## Abstract

Vagal circuits-α7 nAChR (α7 nicotinic acetylcholine receptor, coded by *Chrna7*) signaling utilizes spleen as a hub to dampen systemic inflammatory responses. Vagal innervations also extend to the distal airways and alveoli. Vagotomy and deficiency of α7 nAChR deteriorate *E. coli* and lipopolysaccharide (LPS)-induced acute lung inflammatory responses; however, the underlying mechanisms remain elusive. Here, we hypothesized that vagal circuits would limit splenic release and lung recruitment of α7 nAChR^+^CD11b^+^ cells (CD11b is coded by *Itgam*, a surface marker of monocytes and neutrophils) via phosphorylation of AKT1 and that this process would define the severity of lung injury. Using both *E. coli* and LPS-induced lung injury mouse models, we found that vagotomy augmented splenic egress and lung recruitment of α7 nAChR^+^CD11b^+^ cells, and consequently worsened lung inflammatory responses. Rescue of vagotomy with an α7 nAChR agonist preserved α7 nAChR^+^CD11b^+^ cells in the spleen, suppressed recruitment of these cells to the lung and attenuated lung inflammatory responses. Vagal signals via α7 nAChR promoted serine473 phosphorylation of AKT1 in α7 nAChR^+^CD11b^+^ cells and stabilized these cells in the spleen. Deletion of *Akt1 *enhanced splenic egress and lung recruitment of α7 nAChR^+^CD11b^+^ cells, which elicited neutrophil-infiltrated lung inflammation and injury. Vagotomy and double deletion of *Chrna7* and *Itgam* reduced serine473 phosphorylation of AKT1 in the spleen and BAL (bronchoalveolar lavage) Ly6C^int^Gr1^hi^ neutrophils and Ly6C^hi^ monocytes, and they facilitated the recruitment of neutrophils and monocytes to the airspaces of *E. coli-*injured lungs. Double deletion of *Chrna7* and *Itgam* increased lung recruitment of monocytes and/or neutrophils and deteriorated *E. coli* and LPS-induced lung injury. Thus, signals of vagal circuits engaging with AKT1 in α7 nAChR^+^CD11b^+^ cells attenuate *E. coli* and LPS-induced acute lung inflammatory responses. Targeting this signaling pathway could provide novel therapeutic strategies for treating acute lung injury.

## Introduction

Acute respiratory distress syndrome (ARDS), characterized by acute lung injury (ALI), has a mortality of 30–40%, even if the patients were supported by advanced intensive care [1]. Pneumonia, severe sepsis and acid aspiration are the most serious causes of ARDS [1–3]. Gram-negative sepsis derived ALI is characterized by neutrophil alveolitis and increased permeability of the lung microvascular endothelial and alveolar epithelial barriers [4–6]. Recruitment of monocytes and neutrophils to the lung is a key event in the propagation of lung inflammation and injury [7, 8].

Cellular and humoral factors could modulate inflammatory responses; however, recent investigations have demonstrated that the inflammatory reflex could regulate innate immune responses as well [9–12]. The vagus nerve, the cranial nerve X, originates from the medullar oblongata, which consists of four nuclei: dorsal nucleus, nucleus ambiguous, nucleus tractus solitarius (NTS) and spinal nucleus of trigeminal nerve [13, 14]. The vagus nerve includes both sensory (afferent) and motor (efferent) fibers [15]. About 80% of the afferent sensory fibers are contained in the vagus nerve and are responsible for transmission of the information to the NTS [13]. The afferent sensory neural arc detects the molecular products of injury, infection and inflammation [16]. The efferent arc transmits action potentials from the NTS to the periphery by the vagus nerve. This process culminates in the release of the neurotransmitter acetylcholine, which interacts with innate immune cells that express the α7 subunit of nicotinic acetylcholine receptor (α7 nAChR) [16]. The afferent and efferent arcs, the information integrating center in the central nervous system, and α7 nAChR-expressing immune cells form an inflammatory reflex [16], which provides the host with a fast, discrete and localized means of controlling the immune responses [9, 17].

It should be emphasized that the vagus nerve ramifies in the celiac ganglion giving rise to the postganglionic splenic nerve that terminates in the spleen. Splenic nerve endings release norepinephrine that activate β2 AR (adrenergic receptor)-expressing T memory lymphocytes (CD4^+^CD44^high^CD62L^low^) or CD4^+^CHAT^+^ (choline acetyltransferase) cells and initiate synthesis of acetylcholine (ACh) [18]. ACh could activate splenic α7 nAChR-expressing macrophages to inhibit NF-kB activity and dampen proinflammatory cytokine production (especially TNF-α) [18–22]. Therefore, the spleen is the hub of efferent arc of vagal circuits, which forms the cholinergic anti-inflammatory pathway (CAP) [23, 24].

The vagus nerve innervates the distal airway of the lung, especially in the alveoli [25, 26], where sensors or pathogen recognition receptors in the vagal afferent arc may locate. Some airway sensors are predominantly innervated by vagal afferent fibers derived from the nodose ganglion [27]. The information of lung infection and inflammation is transmitted via the afferent arm to the NTS. After integration, the pulmonary vagal nerve endings secrete ACh that can activate α7 nAChR-expressing proinflammatory cells, suppress NF-kB activation and proinflammatory cytokines, and therefore lessen the extent of lung inflammation and injury [28, 29]. Vagotomy and deficiency of α7 nAChR worsen lipopolysaccharide (LPS) or *Escherichia coli *(*E. coli*)-induced ALI [28, 29]. Thus, pulmonary parasympathetic inflammatory reflexes modulate lung infection and immunity [9, 30, 31].

Vagal circuits could influence trafficking of inflammatory cells via CD11b in the spleen [32]. CD11b is required for adhesion, trafficking, chemotaxis and phagocytosis of proinflammatory cells [33–40]. Toll-like receptor (TLR)-triggered activation of CD11b integrin inhibits TLR signaling in innate immune responses [41]. AKT1 (serine-threonine protein kinase) is a part of PI3K-dependent signaling pathway involved in multiple cellular responses including survival, growth proliferation and migration [42]. Particularly, AKT1 signaling negatively regulates neutrophil recruitment and activation in ALI [43]. Lung α7 nAChR-expressing CD11b^+^ and Gr1^+^ cells are significantly increased during *E. coli* lung infection [29]; but the sources of these cells are elusive. Moreover, the spleen is not only a hub of CAP [18, 23, 24] but also a reservoir of proinflammatory cells (especially, monocytes) [44]. Therefore, we tested whether vagal circuits would regulate spleen release and lung recruitment of α7 nAChR^+^CD11b^+^ cells via phosphorylation of AKT1 by which determined the severity of lung injury.

In this study, we found that disruption of vagal circuit signals promoted splenic release and lung recruitment of α7 nAChR^+^CD11b^+^ cells and reduced *E. coli* and LPS-challenged lung injury. Administration of α7 nAChR agonist PHA568487 to *E. coli* and LPS-challenged vagotomized mice stabilized splenic α7 nAChR^+^CD11b^+^ cells by enhancing phosphorylation of AKT1, reduced lung recruitment of this cell population, and therefore mitigated the severity of lung injury. Deletion of *Akt1* enhanced discharge of splenic α7 nAChR^+^CD11b^+^ cells and lung recruitment of these cells and worsened *E. coli* and LPS-induced lung injury. Double deletion of *Chrna7* and *Itgam* reduced splenic CD4^+^CHAT^+^ cells and phosphorylation of AKT1 in splenic and BAL ly6C^hi^ monocytes and neutrophils, augmented recruitment of these proinflammatory cells to LPS and *E. coli*-challenged lungs, and worsened lung injury. Our findings provide insight into vagal-immune cell regulation of lung inflammatory responses and implicate a therapeutic target for acute lung inflammatory injury.

## Results

### Disruption of vagal circuits boosts spleen egress and lung recruitment of granulocytes and worsens LPS-induced lung injury

**Figure 1:**
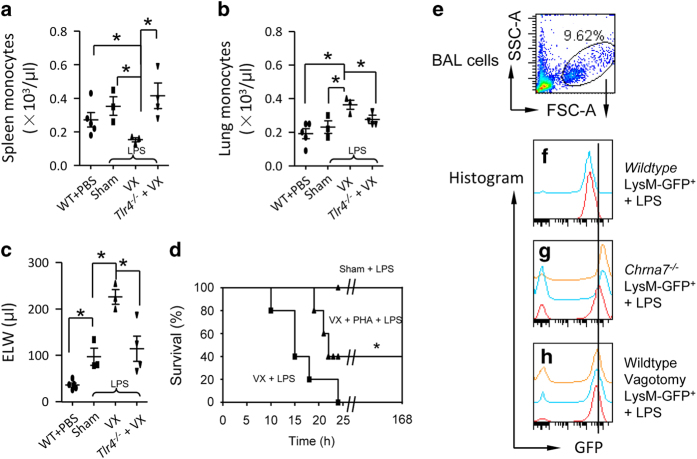
Disruption of vagal circuits exacerbates LPS-induced ALI by enhancing egress of splenic monocytes and migration of granulocytes towards the injured lungs. (**a**–**c**) Effect of vagotomy on splenic and pulmonary monocytes, and extravascular lung water (ELW) in LPS-induced ALI. The mice were divided into four groups: WT (PBS IT), sham+LPS (5 mg kg^−1^ IT), vagotomy+LPS (5 mg kg^−1^ IT) or Tlr4^−/−^+vagotomy+LPS (5 mg kg^−1^ IT). Mice were killed at 15 h after intratracheal LPS to measure splenic monocytes (**a**), pulmonary monocytes (**b**) and ELW (**c**). *N*=3–5 in each group. Data were pooled from three experiments. *P<0.05, one-way ANOVA with Bonferroni *post hoc* test. Data are presented as mean±s.d. (**d**) Effect of vagotomy on mortality of LPS-induced ALI. The mice were divided into three groups: sham+LPS (*n*=8), vagotomized+LPS (*n*=9) and vagotomized+PHA568487+LPS (*n*=4). The mice were IT instilled with LPS at dose of 5 mg kg^−1^. Mice of the vagotomized+PHA568487+LPS group were given an intraperitoneal (ip) injection of PHA568487 (0.8 mg kg^−1^) 15 min before LPS, and repeated the same dose 6, 12 and 18 h, respectively, after LPS to maintain drug concentration in the blood. The corresponding vehicles were given in the other two groups. The mortality of mice was followed up for 7 days. *P<0.05 by log-rank test. (**e**–**h**) Effect of deficiency of α7 nAChR and vagotomy on LysM-GFP^+^ cell migration to the airspaces of the lung during LPS-induced ALI. The mice were divided as follows: wildtype LysM-GFP^+^ mice receiving an IT of LPS (5 mg kg^−1^), *Chrna7*^−/−^LysM-GFP^+^ mice receiving an IT of *E. coli* (5 mg kg^−1^), and vagotomized wildtype LysM-GFP^+^ mice receiving an IT of LPS (5 mg kg^−1^). The mice were killed at 24 h after LPS challenge. The BAL cells were collected for flow cytometry. The whole-cell population was gated (**e**). The LysM-GFP histogram was applied to each sample in wildtype LysM-GFP^+^+LPS (*n*=2) (**f**), Chrna7^−/−^LysM-GFP^+^+LPS (*n*=3) (**g**) and vagotomized LysM-GFP^+^+LPS (*n*=3) (**h**).

As the hub of CAP [23, 24], spleen is also a reservoir of monocytes, and these cells can be deployed to the inflammatory sites during injury [44]. To test whether the disruption of vagal signals could reduce monocytes in the spleen, while increasing monocytes in the lung in an LPS-induced lung injury mouse model, we unilaterally excised the right cervical vagus nerve of mice and then intratracheally (IT) challenged them with LPS. 15 h later, we found that after LPS challenge, vagotomy enhanced the deployment of splenic monocytes (Figure 1a), accumulation of these cells in the lung (Figure 1b) and elevation of extravascular lung water (Figure 1c) compared with the sham mice, which received the same procedures as vagotomy, but the vagus remained intact. Deletion of *Tlr4* in LPS-challenged vagotomized mice reversed these effects (Figure 1a–c), suggesting that vagal signals negatively regulate TLR4 signaling. To show that vagal signals protected LPS-challenged mice via α7 nAChR, we followed up with the LPS-challenged sham and vagotomized mice, and PHA568487 (PHA)-treated LPS-challenged vagotomized mice for 7 days. We found that LPS-challenged sham mice survived for 7 days after LPS; however, the LPS-challenged vagotomized mice died within 25 h. Supplementing with α7 nAChR agonist PHA568487 could rescue 40% of LPS-challenged vagotomized mice during 7 day observation (Figure 1d). In LPS-challenged vagotomized mice, neutrophils were decreased in the spleen (Supplementary Figure S1A), and increased in the peripheral blood (Supplementary Figure S1B) and lungs (Supplementary Figure S1C; MPO, an index of neutrophil infiltration), where pulmonary edema was exacerbated (Supplementary Figure S1D). The p-P65 NF-κB was increased; however, the p-AKT1^Ser473^ was decreased in the nuclear extract of isolated splenic neutrophils from the LPS-challenged vagotomized mice compared to that in the LPS-challenged sham mice (Supplementary Figure S1E and F). These findings indicate that splenic neutrophil p-AKT1^Ser473^ might be a negative regulator for lung inflammatory responses. We confirmed that rabbit anti-α7 nAChR antibody could bind α7 nAChR specifically by comparing α7 nAChR^+^ cells between wildtype and *Chrna7*^−/−^ lung, bone marrow (BM) and spleen cells. Fluor-633 α-bungarotoxin labeling in BM cells also proved the specificity of this antibody (Supplementary Figure S2A–G). In the non-LPS challenge condition, vagotomy did not affect percentage of monocytes (Ly6C^hi^Ly6G^int^) and neutrophils (Ly6C^int^Ly6G^*hi*^) in the spleen, BM and lung (Supplementary Figure S3A–J).

We used LPS-challenged LysM-GFP^+^ mice (GFP-positive cells are granulocytes [45]) to detect whether vagotomy or deficiency of α7 nAChR could facilitate the recruitment of granulocytes to inflammatory sites. By flow cytometry gating whole BAL cells (Figure 1e), we found that LysM-GFP^+^ granulocytes in the BAL were increased in LPS-challenged LysM-GFP^+^ *Chrna7*^*−/−*^ or LPS-challenged LysM-GFP^+^ vagotomized mice compared to LPS-challenged LysM-GFP^+^ sham mice at 24 h (Figure 1f–h), suggesting that vagal-α7 nAChR signaling controls the recruitment of granulocytes towards LPS-injured lungs. These findings indicate that vagal signals via α7 nAChR might limit the recruitment of granulocytes to LPS-injured lung and attenuate magnitude of lung injury.

### Vagal signals restrain migration of α7 nAChR^+^CD11b^+^ or Gr1^+^ granulocytes towards LPS-injured lung

**Figure 2:**
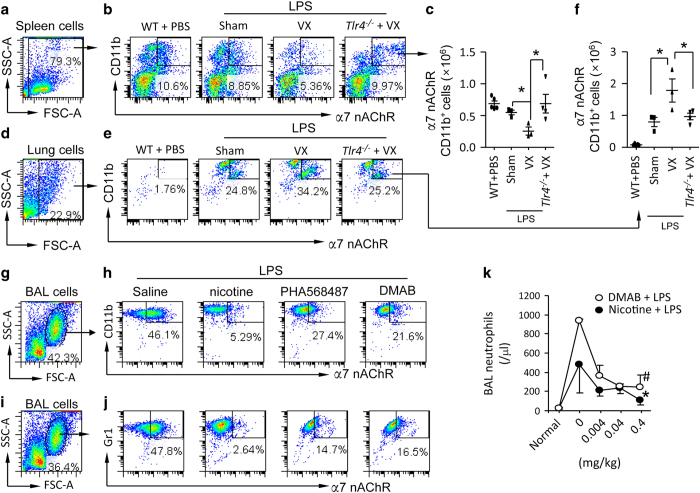
Effect of disruption of vagal circuits and α7 nAChR agonists on α7 nAChR^+^CD11b^+^cells in the spleen and lung in LPS-induced ALI mouse model. (**a**–**f**) Vagotomy affects distribution of α7 nAChR^+^CD11b^+^cells in the spleen and lung in LPS-induced ALI. The mice were divided into four groups: WT (PBS IT), sham+LPS (5 mg kg^−1^ IT), vagotomy+LPS (5 mg kg^−1^ IT) or Tlr4^−/−^+vagotomy+LPS (5 mg kg^−1^ IT). Mice were killed at 15 h after LPS instillation. Spleens and lungs were removed to isolate single cells for flow cytometry. The spleen and lung cells were labeled with PE-CD11b and Fluro-488 α7 nAChR antibodies. The whole live cell population was gated (**a**, spleen; **d**, lung). The number of spleen (**b**) and lung (**e**) α7 nAChR^+^CD11b^+^cells was calculated by percentage of cell population multiplied by total cell counts in each spleen or lung (**c**,** f**). *P<0.05, between labeled groups. *N*=3–4 in each group. One-way ANOVA with Bonferroni *post hoc* test. Data are presented as mean±s.d. (**g**–**j**) Effect of α7 nAChR agonists on the population of α7 nAChR^+^granulocytes in the BAL from LPS-induced ALI. Mice were intraperitoneally pretreated with saline, nicotine (0.4 mg kg^−1^), PHA568487 (a specific α7 nAChR agonist, 0.4 mg kg^−1^) or DMAB (a partial α7 nAChR agonist, 0.4 mg kg^−1^) 15 min before IT of LPS 5 mg kg^−1^. The saline and α7 nAChR agonists were given every 6 h after LPS. The mice were killed at 24 h after LPS to collect BAL cells for flow cytometry. The BAL cells were labeled with Fluor488-α7 nAChR and PE-CD11b or Gr1 antibody. The whole-cell population was selected (**g**, **i**) for analyzing α7 nAChR^+^CD11b^+^cells (**h**) or α7 nAChR^+^Gr1^+^cells (**j**) in the BAL from each group. (**k**) Effect of nicotine and DMAB on BAL neutrophils in LPS-induced ALI. Mice were intraperitoneally pretreated with saline, nicotine (0.004, 0.04 and 0.4 mg kg^−1^) or DMAB (0.004, 0.04 and 0.4 mg kg^−1^) (q6h thereafter, ip) and then received an IT of LPS 5 mg kg^−1^. The mice were killed at 24 h after LPS to collect BAL cells for measurements. *N*=3–4 in each group. *P<0.05, ^#^*P*<0.01, compared with saline-treated group. Data was pooled from two independent experiments. One-way ANOVA with Bonferroni *post hoc* test. Data are presented as mean±s.d.

Splenic α7 nAChR-expressing macrophages can be activated by signals from vagus nerve circuit [9, 10, 18]. Granulocytes express CD11b or Gr1 (marker for neutrophils) [33, 44]. To test whether vagal signals regulate splenic egress and lung recruitment of α7 nAChR^+^CD11b^+^ cells [18], we utilized flow cytometry (Figure 2a, b, d and e). to analyze quantity of α7 nAChR^+^CD11b^+^ cells in the spleen and lung from LPS-induced ALI mice. At 15 h, we found that vagotomy reduced α7 nAChR^+^CD11b^+^ cells in the spleen (Figure 2c), but increased these cells in LPS-challenged lungs (Figure 2f) compared to LPS-challenged sham mice. Deletion of *Tlr4 in* LPS-challenged vagotomized mice could invert these events (Figure 2c and f), suggesting that vagal signals regulate flux of α7 nAChR^+^CD11b^+^ cells during LPS-induced ALI depending on TLR4 signaling. To test whether activation of α7 nAChR would mitigate migration of α7 nAChR^+^CD11b^+^ or α7 nAChR^+^Gr1^+^ cells towards LPS-injured airspaces of the lungs, we pretreated LPS-challenged mice with either saline or α7 nAChR agonists: nicotine, PHA568487 or DMAB. At 24 h, we collected BAL and gated granular cells (Figure 2g and i) and found that the percentage of α7 nAChR^+^CD11b^+^ and α7 nAChR^+^Gr1^+^ cells in the BAL from α7 nAChR agonists-pretreated LPS-challenged mice was reduced compared to saline-pretreated LPS-challenged mice (Figure 2h and j). We pretreated mice with different doses of nicotine or DMAB, and then IT challenged them with LPS. At 24 h, BAL neutrophils were reduced dose-dependently in nicotine or DMAB-treated mice, suggesting that activation of α7 nAChR could limit migration of neutrophils towards the inflamed lungs (Figure 2k). These findings indicate that vagal signals attenuate LPS-induced lung inflammatory responses by limiting lung recruitment of α7 nAChR^+^CD11b^+^ or Gr1^+^ granulocytes.

### Vagal signals lessen lung *E. coli*-induced inflammatory responses by reducing spleen egress and lung recruitment of granulocytes

**Figure 3:**
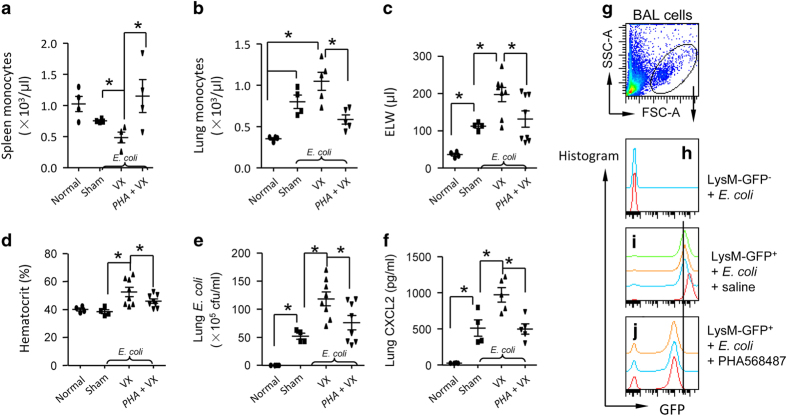
Disruption of vagal circuits exacerbates *E. coli*-induced ALI by enhancing migration of granulocytes towards the injured lungs. (**a**–**d**) Vagal-α7 nAChR signaling regulates quantity of splenic and lung monocytes, pulmonary edema, severity of lung infection and chemokine levels in *E. coli *pneumonia. Mice were randomly divided into four groups: Normal, sham+ *E. coli*, vagotomized+*E. coli* and vagotomized+PHA568487 (PHA)+*E. coli*. Normal controls were naive mice. Sham+*E. coli* and vagotomized+*E. coli* received an IT of *E. coli* (2.5×10^6^) and corresponding vehicle treatment. The vagotomized+PHA+*E. coli *group received an IP of PHA (0.4 mg kg^−1^) 15 min prior to intratracheal *E. coli* (2.5×10^6^). The PHA therapy was given IP every 6 h. The mice were killed 24 h later to measure number of splenic (**a**) and lung monocytes (**b**), ELW (**c**), hematocrit (**d**), *E. coli* colonies (**e**) and CXCL2 (a chemokine of neutrophils) (**f**) in the supernatants of lung homogenates. *N*=4–8 in each group. *P<0.05, compared with the indicated group. Data was pooled from two independent experiments. (**g**–**j**) Effect of activation of α7 nAChR on migration of granulocytes towards the injured lungs during *E. coli* pneumonia. The mice were divided as follows: LysM-GFP^−^ mice receiving an IT of *E. coli* (2.5×10^6^ cfu), LysM-GFP^+^ mice receiving an IT of *E. coli* (2.5×10^6^ cfu), and LysM-GFP^+^ mice pretreated with PHA568487 (a specific α7 nAChR agonist, 0.4 mg kg^−1^, given IP every 6 h), and received an IT of *E. coli* (2.5×10^6^ cfu). The mice were killed at 24 h after *E. coli* infection. The BAL cells were collected for flow cytometry. The whole-cell population was gated (**g**). The LysM-GFP histogram was applied to each sample in LysM-GFP^−^+ *E. coli* (*n*=2) (**h**), LysM-GFP^+^+*E. coli* (*n*=4) (**i**) and LysM-GFP^+^+*E. coli*+PHA568487 (*n*=3) (**j**).

To determine if vagal signals could lessen *E. coli*-induced lung inflammatory responses by reducing splenic egress and lung recruitment of granulocytes, we divided mice into four groups of mice: normal, sham+*E. coli*, vagotomy+*E. coli* and PHA568487-pretreatment+vagotomy+*E. coli*. The mice were killed at 24 h after IT challenge of *E. coli*. We found that vagotomy decreased monocytes in the spleen (Figure 3a) and elevated these cells in *E. coli*-infected lungs (Figure 3b). Pulmonary edema (Figure 3c), hematocrit (an index of systemic permeability) (Figure 3d), lung *E. coli* colonies (Figure 3e) and CXCL2 levels in supernatant of lung homogenate were all elevated (Figure 3f) in *E. coli*-challenged vagotomized mice compared to *E. coli*-challenged sham mice. However, these effects of vagotomy could be reversed by applying α7 nAChR agonist PHA568487 to the vagotomized animals (Figure 3a–f), suggesting that vagal signals control the flux of splenic monocytes and lung bacterial-induced inflammatory responses dependent on α7 nAChR activation. To further prove that activation of α7 nAChR could reduce the recruitment of granulocytes to *E. coli*-infected lungs, we collected BAL cells from LysM-GFP^−^+*E. coli*, saline-pretreated LysM-GFP^+^+*E. coli*, and PHA568487-pretreated LysM-GFP^+^+*E. coli* groups. At 24 h, we gated live BAL cells (Figure 3g) and found that BAL GFP^+^ granulocytes were increased in saline-pretreated LysM-GFP^+^+*E. coli* group compared to LysM-GFP^−^+*E. coli* group (Figure 3h and i). However, administration of α7 nAChR agonist PHA568487 in LysM-GFP^+^+*E. coli* group reduced BAL GFP^+^ granulocytes compared to saline-pretreated LysM-GFP^+^+*E. coli* group (Figure 3i and j), supporting the conclusion that vagal signals reduce lung recruitment of granulocytes in *E. coli*-challenged lungs via α7 nAChR activation.

### Vagal signals via serine473 phosphorylation of AKT1 limit discharge of α7 nAChR^+^CD11b^+^ cells from spleen and suppress recruitment of these cells to the injured lung

**Figure 4:**
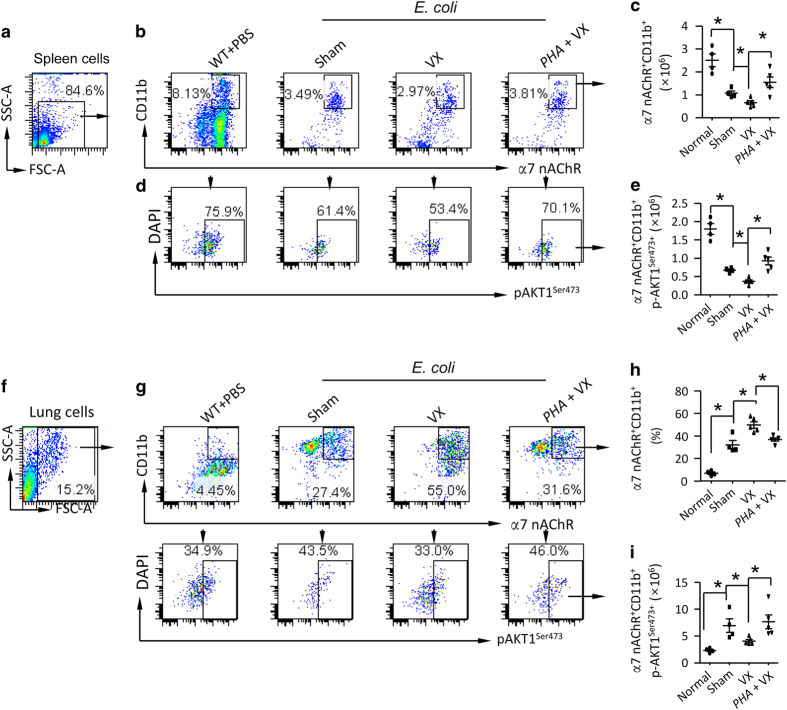
Vagal-α7 nAChR signaling engages with phosphorylation of AKT1^Ser473+^ to prevent egress of α7 nAChR^+^CD11b^+^ cells from spleen and recruitment of these cells towards *E. coli*-injured lungs. (**a**–**e**) Vagal-α7 nAChR signaling regulates of p-AKT1^Ser473+^in splenic α7 nAChR^+^CD11b^+^cells during*** **E. coli *pneumonia. Sham, vagotomized and vagotomized plus PHA568487 (0.4 mg kg^−1^ pretreated 15 min prior to *E. coli* (2.5×10^6^ cfu) and given every 6 h, IP). The mice were killed 24 h after infection. The spleen cells were collected and labeled with the indicated fluorescent antibodies. The granular cells were gated (**a**). The α7 nAChR^+^CD11b^+^ cells were first analyzed and then calculated the percentage of p-AKT1^Ser473+^ cells (**b**–**e**). *N*=4–5 in each group. *P<0.05, one-way ANOVA with Bonferroni *post hoc* test. (**f**–**i**) Flow cytometry analysis of α7 nAChR^+^CD11b^+^ cell population the lung during*** **E. coli *pneumonia. Using the same experimental setting as (**a**–**e**), the lung cells were collected and labeled with the indicated fluorescent antibodies. The granular cells were gated (**f**). The α7 nAChR^+^CD11b^+^ cells and α7 nAChR^+^CD11b^+^p-AKT1^Ser473+^ cells were analyzed (**g**). The percentage of α7 nAChR^+^CD11b^+^ cells and number of α7 nAChR^+^CD11b^+^p-AKT1^Ser473+^ cells were calculated (**h**, **i**). *N*=4–5 in each group. *P<0.05, one-way ANOVA with Bonferroni *post hoc* test. Data are presented as mean±s.d.

We performed lung immunofluorescence and found that α7 nAChR^+^ and α7 nAChR^+^Gr1^+^ cells were increased in *E. coli*-infected vagotomized mice compared to *E. coli*-infected sham mice 12 and 24 h after IT challenge of *E. coli* (Supplementary Figure S4A and B). This finding supports that vagal signals regulate recruitment of α7 nAChR^+^ granulocytes to *E. coli*-infected lung. To clarify the underlying mechanism that defines the movement of α7 nAChR^+^ granulocyte, we isolated spleen, peripheral blood and lung cells from normal, sham+*E. coli*, vagotomy+*E. coli*, and PHA568487-pretreatment+vagotomy+*E. coli* mice to analyze α7 nAChR^+^CD11b^+^ cells and serine473 phosphorylation of AKT1 24 h after *E. coli* challenge (Figure 4a, b and d). We found that splenic α7 nAChR^+^CD11b^+^ cells were markedly decreased in *E. coli*-infected vagotomized mice compared with *E. coli*-infected sham mice (Figure 4c); furthermore, p-AKT1^*Ser473+*^α7 nAChR^+^CD11b^+^ cells were declined in *E. coli*-infected vagotomized mice compared to *E. coli*-infected sham mice (Figure 4e). The above changes were reversed if administrating α7 nAChR agonist PHA568487 to the vagotomized mice (Figure 4c and e). These findings indicate that serine473 phosphorylation of AKT1 in α7 nAChR^+^CD11b^+^ cells suppress exit of these cells from spleen. By separately analyzing lymphocyte (L), monocyte (M) and polymorphonuclear leukocyte gates (Supplementary Figure S5A, D and G) in peripheral blood cells from these four groups of mice (Supplementary Figure S5B, E and H), we found that there were more blood α7 nAChR^+^CD11b^+^ cells in *E. coli*-infected vagotomized mice than *E. coli*-infected sham mice; however, this change was counteracted by activation of α7 nAChR (Supplementary Figure S5C, F and I). We gated the α7 nAChR^+^CD11b^+^ population from lung cells (Figure 4f and g) and found that α7 nAChR^+^CD11b^+^ cells were increased in *E. coli*-infected vagotomized lung compared to relative to *E. coli*-infected sham lung; administrating α7 nAChR agonist PHA568487 corrected this change (Figure 4h). The number of lung p-AKT1^*Ser473+*^α7 nAChR^+^CD11b^+^ cells was reduced in *E. coli*-infected vagotomized mice compared with *E. coli*-infected sham mice, and activation of α7 nAChR in *E. coli*-infected vagotomized mice restored this change (Figure 4i). The findings indicate that vagal-α7 nAChR signal via phosphorylation of AKT1 at serine473 site stabilizes α7 nAChR^+^CD11b^+^ cells in the spleen and prevents these cells from migrating to peripheral blood and *E. coli*-infected lung. To demonstrate that activation of α7 nAChR induces AKT1 phosphorylation, which suppresses proinflammatory responses, we pretreated wildtype and *Chrna7*^−/−^ splenic neutrophils with α7 nAChR specific agonist PHA568487 and then challenged them with LPS and separated cytoplasm and nucleus 1 h later. Activation of α7 nAChR promoted phosphorylation of p-AKT1 at Ser473 site in splenic neutrophil cytoplasm (Supplementary Figure S6A), but reduced it in the nucleus (Supplementary Figure S6B). Activation of α7 nAChR also suppressed CXCL2 production in LPS-challenged splenic neutrophils (Supplementary Figure S6C). Inhibition of phosphorylation of AKT1 by Wortmannin increased CXCL2 production in LPS-challenged splenic neutrophils (Supplementary Figure S6D). Deletion of *Itgam* increased CXCL2 and TNF-α production in LPS-challenged splenic neutrophils (Supplementary Figure S6E). These findings provide evidence that α7 nAChR-p-AKT1 signaling negatively regulates proinflammatory responses in neutrophils.

### Deletion of Akt1 enhances spleen egress and lung recruitment of α7 nAChR^+^CD11b^+^ cells and deteriorates lung injury

**Figure 5:**
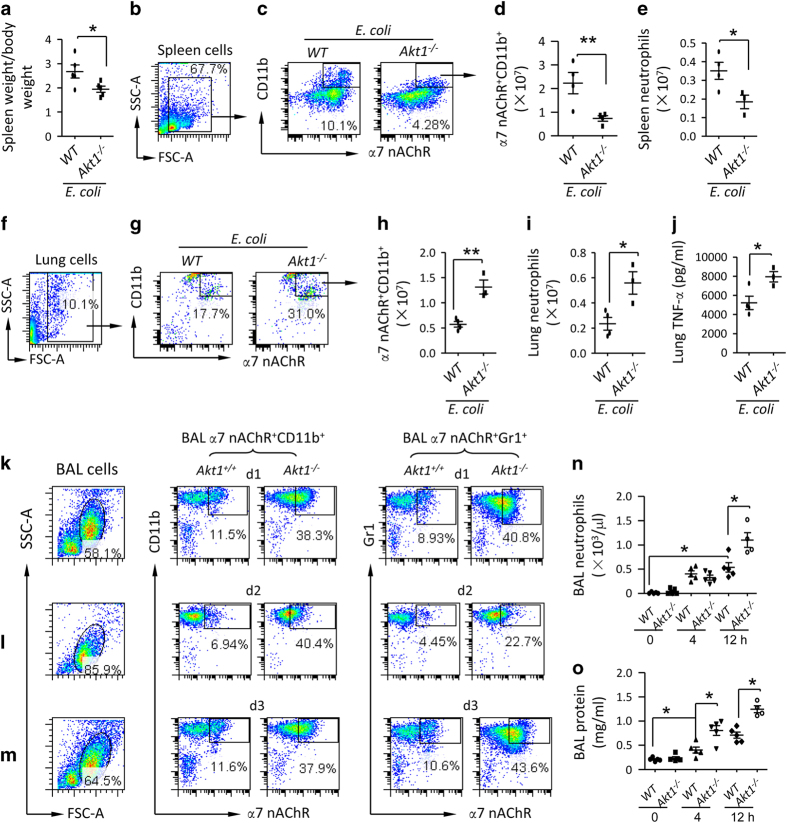
Deficiency of AKT1 facilitates release of α7 nAChR^+^CD11b^+^ cells from spleen and recruitment of these cells to the injured lungs. (**a**–**e**) Deficiency of AKT1 reduces splenic α7 nAChR^+^CD11b^+^ cells. The wildtype and Akt1^−/−^ mice were challenged with an IT of *E. coli* (2.5×10^6^ cfu), and were killed 24 h later. The spleen/body weight ratio was compared (**a**). The spleen cells were labeled with anti-α7 nAChR and CD11b fluorescent antibodies. The whole-cell population was gated to analyze α7 nAChR^+^CD11b^+^ cell population (**b**,** c**). The percentage multiplied counts of splenocytes to obtain absolute number of α7 nAChR^+^CD11b^+^ cells (**d**). Spleen neutrophils were counted by Wright’s staining (**e**). *N*=3–4 in each group. *P<0.05, **P<0.01, Student’s *t*-test. Data are presented as mean±s.d. (**f**–**j**) Deficiency of AKT1 enhances recruitment of α7 nAChR^+^CD11b^+^ cells and neutrophils to the lung and lung TNF-α level. The lung cells were labeled with anti-α7 nAChR and CD11b fluorescent antibodies. The whole-cell population was gated to analyze α7 nAChR^+^CD11b^+^ cell population (**f**,** g**). The percentage multiplied counts of splenocytes to obtain absolute number of α7 nAChR^+^CD11b^+^ cells (**h**). Lung neutrophils were counted by Wright’s staining (**i**) and TNF-α levels were measured by ELISA (**j**). *N*=3–4 in each group. *P<0.05, Student’s *t*-test. Data are presented as mean±s.d. (**k**–**m**) Effect of deficiency of Akt1 on α7 nAChR^+^CD11b^+^ and α7 nAChR^+^Gr1^+^ cell population in the BAL during*** **E. coli *pneumonia. The wildtype and Akt1^−/−^ mice were challenged with an IT of E. coli (2.5×10^6^ cfu). The mice were killed at d 1, 2 and 3 after *E. coli* challenge. The BAL was collected to harvest BAL cells for flow analysis. The cells were labeled with anti-α7 nAChR, CD11b and Gr1 fluorescent antibodies. The whole-cell population was gated. α7 nAChR^+^CD11b^+^ and α7 nAChR^+^Gr1^+^ cells were analyzed at d 1(**k**), d 2(**l**) and d 3(**m**) in the BAL cells. (**n**,** o**) Effect of deficiency of AKT1 on BAL neutrophils and protein in LPS-induced ALI. Wildtype and Akt1-deleted mice were IT challenged with LPS (5 mg kg^−1^). The mice were killed at 4 and 12 h after LPS challenge. BAL was collected to measure neutrophil count (**n**) and protein levels (**o**). *N*=4–5 in each group. *P<0.05, Student’s *t-*test. Data are presented as mean±s.d.

To investigate whether deletion of *Akt1* could enhance splenic release of α7 nAChR^+^CD11b^+^ cells, we isolated spleen cells from *E. coli-*infected wildtype and *Akt1*^−/−^ mice 24 h after IT *E. coli*. The spleen/body weight ratio in *E. coli*-infected *Akt1*^−/−^ mice was lower than *E. coli*-infected wildtype mice (Figure 5a). We gated α7 nAChR^+^CD11b^+^ cell population in spleen cells (Figure 5b and c) and found that the number of splenic α7 nAChR^+^CD11b^+^ cells was lower in *E. coli*-infected *Akt1*^−/−^ mice than *E. coli*-infected wildtype mice (Figure 5d). The number of spleen neutrophils was also lower in *E. coli*-infected *Akt1*^−/−^ mice (Figure 5e). In lung cells (Figure 5f), percentage (Figure 5g) and absolute number (Figure 5h) of α7 nAChR^+^CD11b^+^ cells were higher in *E. coli*-infected *Akt1*^−/−^ mice than *E. coli*-infected wildtype mice. Lung neutrophils (Figure 5i) and TNF-α (Figure 5j) in *E. coli*-infected *Akt1*^−/−^ mice were elevated compared to *E. coli*-infected wildtype mice. To study whether deletion of *Akt1* would facilitate recruitment of α7 nAChR^+^CD11b^+^ and α7 nAChR^+^Gr1^+^ cells to *E. coli*-infected lung, we IT challenged wildtype and *Akt1*^−/−^ mice with *E. coli* and killed them at d1, d2 and d3. The BAL cells were isolated to analyze α7 nAChR^+^CD11b^+^ and α7 nAChR^+^Gr1^+^ cells (Figure 5k–m). The percentage of BAL α7 nAChR^+^CD11b^+^ and α7 nAChR^+^Gr1^+^ cells was increased in *E. coli*-infected *Akt1*^−/−^ mice compared to *E. coli*-infected wildtype mice (Figure 5k–m). In an LPS-induced ALI mouse model, BAL neutrophils and protein levels were higher in LPS-challenged *Akt1*^−/−^ mice compared to LPS-challenged wildtype mice (Figure 5n and o). We also IT challenged wildtype and *Akt1*^−/−^ mice with *E. coli* to study BAL profiles. At 24 h, we found that BAL protein (Supplementary Figure S7A), *E. coli* colonies (Supplementary Figure S7B) and TNF-α (Supplementary Figure S7C) were higher in *E. coli*-infected *Akt1*^−/−^ mice than *E. coli*-infected wildtype mice. The findings strongly support that deletion of *Akt1* enhances splenic release of α7 nAChR^+^CD11b^+^ cells, facilitates migration of these cells towards *E. coli* and LPS-challenged lung, and propagates lung injury.

### Deletion of Chrna7 reduces phosphorylation of AKT1^Ser473^ which facilitates splenic egress and lung recruitment of CD11b^+^ cells towards *E. coli*-infected lung

**Figure 6:**
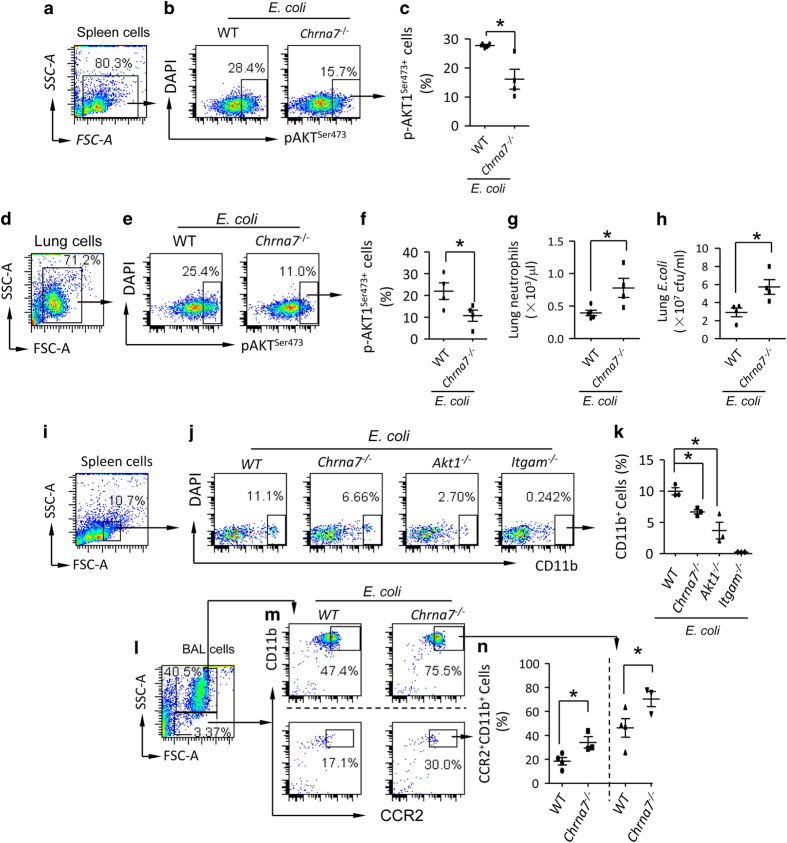
Deficiency of α7 nAChR enhances release of CD11b^+^ cells from spleen and recruitment of these cells towards the injured lungs by reducing phosphorylation of AKT1^Ser473+^. (**a**–**f**) Deletion of *Chrna7* reduces serine phosphorylation of AKT1 in the spleen and lung cells during* E. coli *pneumonia. Wildtype and *Chrna7*-deleted mice were IT challenged with *E. coli* (2.5×10^6^ cfu). The mice were killed at 24 h after *E. coli *challenge. The spleen (**a**) and lung (**d**) cells were isolated and labeled with PE-anti-p-AKT1^Ser473^ antibody after permeabilization. The whole-cell population was gated (**b**,** e**). p-AKT1^Ser473+^cells were analyzed (**c**,** f**). The percentage of p-AKT1^Ser473+^cells was presented. *N*=4 in each group, *P<0.05, Student’s *t*-test. Data are presented as mean±s.d. (**g**, **h**) Deletion of *Chrna7* affects lung neutrophils and *E. coli *colonies during lung infection. Wildtype and *Chrna7*-deleted mice were IT challenged with *E. coli* (2.5×10^6^ cfu). The mice were killed at 24 h after *E. coli* challenge. Lungs cells were counted by Wright’s staining (**g**). Serial dilution of the supernatant of homogenized lung and bacterial culture was used to detect *E. coli* colonies (**h**). *N*=4 in each group, *P<0.05, Student’s *t*-test. Data are presented as mean±s.d. (**i**–**k**) Depletion of *Chrna7* reduces splenic CD11b^+^cells during *E. coli* pneumonia. The wildtype, *Chrna7*^−/−^, Akt1^−/−^ and Itgam^−/−^ mice were, respectively, challenged with *E. coli* (2.5×10^6^ cfu) intratracheally. The mice were killed at 24 h after E. coli challenge. The spleen cells were isolated and labeled with PE-anti-CD11b antibody. The granular cells were gated (**i**) in each group (**j**). The percentage of CD11b^+^ cells was presented (**k**). *N*=3–4 in each group, *P<0.05. Data are presented as mean±s.d. (**l**–**n**) Depletion of *Chrna7* increases BAL CCR2^+^CD11b^+^cells during*** **E. coli *pneumonia. The wildtype and *Chrna7*^−/−^ mice were respectively challenged with *E. coli *(2.5×10^6^ cfu) intratracheally. The mice were killed at 24 h after *E. coli* challenge. The BAL cells were isolated and labeled with FITC-anti-CCR2 and PE-anti-CD11b antibodies. The larger granular and smaller granular cells were separately gated (**l**,** m**). The percentage of CCR2^+^CD11b^+^ cells was calculated (**n**). *N*=3–4 in each group, *P<0.05, student’s *t*-test. Data are presented as mean±s.d.

In *E. coli* pneumonia, *E. coli-*infected *Chrna7*^−/−^ or vagotomized mice had higher BAL protein (Supplementary Figure S8A), *E. coli* colonies (Supplementary Figure S8B) and TNF-α (Supplementary Figure S8C), and they had less splenic p-AKT1^*Ser473*^, p-STAT3 and p-ERK (Supplementary Figure S8D–F), suggesting that AKT1 is in the downstream of vagal-α7 nAChR signaling. To verify this, we isolated the spleen (Figure 6a and b) and lung cells (Figure 6d and e) from *E. coli*-infected wildtype and *Chrna7*^−/−^ mice. By flow cytometry analysis, we found that the percentage of p-AKT1^*Ser473*^ expressing cells in both the spleen (Figure 6c) and lung (Figure 6f) from *E. coli*-infected *Chrna7*^−/−^ mice was decreased compared to that from *E. coli*-infected wildtype mice. The lower phosphorylation of serine473 AKT1 was associated with higher levels of lung neutrophils (Figure 6g) and *E. coli* colonies (Figure 6h) compared to wildtype. This finding supports the conclusion that α7 nAChR via phosphorylation of AKT1 regulates neutrophil migration and bacterial growth. Next, we tested whether the deletion of *Chrna7* would affect the distribution of CD11b^+^ cells between the spleen and the lung during *E. coli* infection. We separately IT challenged wildtype, *Chrna7*^*−/−*^*, Akt1*^*−/−*^ and *Itgam*^*−/−*^ mice with *E. coli* and isolated splenic cells at 24 h. The granular cells were gated (Figure 6i) and CD11b^+^ cells were subgated in each group (Figure 6j). The percentage of CD11b^+^ cells was decreased in *E. coli*-infected *Chrna7*^*−/−*^ and *Akt1*^*−/−*^ spleens compared to *E. coli*-infected wildtype spleen (Figure 6k), suggesting that α7 nAChR and AKT1 are important for controlling egress of splenic CD11b^+^ cells. We also isolated BAL cells from *E. coli*-infected wildtype and *Chrna7*^*−/−*^ mice at 24 h. The BAL cells were labeled with anti-CCR2 (a chemokine receptor of MCP-1, which has a role in chemotaxis of monocytes) and CD11b fluorescent antibodies. The granular cells were separately gated (Figure 6l) and CCR2^+^CD11b^+^ cells were subgated in wildtype and *Chrna7*^*−/−*^ cells (Figure 6m). The percentage of CCR2^+^CD11b^+^ cells was increased in the *E. coli*-infected *Chrna7*^*−/−*^ BAL compared to *E. coli*-infected wildtype BAL (Figure 6n). These findings support that α7 nAChR via downstream AKT1 regulates spleen egress and lung recruitment of CD11b^+^ cells. Furthermore, we tested whether the deletion of *Akt1* could simulate deletion of *Chrna7* to deteriorate *E. coli* and LPS-induced ALI. We found that ELW (Supplementary Figure S9A), blood neutrophils and monocytes (Supplementary Figure S9B and C), and lung MPO activity (Supplementary Figure S9D) were increased in *E. coli*-infected *Akt1* and *Chrna7*-deleted mice compared with *E. coli*-infected wildtype counterparts. Higher mortality was found in *E. coli*-infected *Akt1* and *Chrna7*-deleted mice compared with *E. coli*-infected wildtype mice (Supplementary Figure S9E). The findings suggest that vagal-α7 nAChR-p-AKT1 signaling negatively regulates egress of splenic CD11b^+^ cells and the severity of *E. coli* and LPS-induced lung inflammatory injury.

### Vagotomy and double deletion of Chrna7 and Itgam reduce splenic ly6C^int^Gr1^hi^ and ly6C^hi^p-AKT1^ser473+^ and CD4^+^CHAT^+^ ACh-producing cells

**Figure 7:**
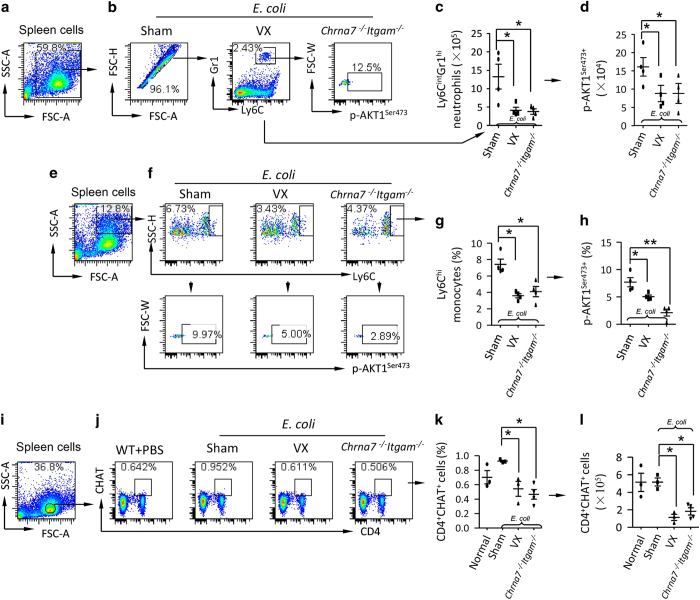
Vagotomy and double deletion of *Chrna7* and *Itgam* reduce p-AKT1^Ser473+^ neutrophils and monocytes and ACh-producing CD4^+^CHAT^+^ cells during* E. coli *pneumonia. (**a**–**d**) Flow cytometry analysis of AKT1^Ser473^phosphorylation in splenic neutrophils. The sham, vagotomized and *Chrna7*^−/−^*Igtam*^−/−^ mice were IT challenged with *E. coli *(2.5×10^6^ cfu). The mice were killed at 24 h after *E. coli* challenge. Spleen cells were collected and labeled with fluorescent antibodies. (**a**) Whole-cell population was gated. (**b**) Ly6C^Int^Gr1^hi^ neutrophils were grouped, then subgated p-AKT1^Ser473+^ cells. (**c**) Number of splenic Ly6C^int^Gr1^hi^ neutrophils. (**d**) Number of p-AKT1^Ser473+^ cells in Ly6C^int^Gr1^hi^ gate. *N*=4 in each group, *P<0.05. Data are presented as mean±s.d. (**e**–**h**) Flow cytometry analysis of AKT1^Ser473^phosphorylation in splenic monocytes. Using the same experimental setting as (**a**–**d**), granular cells were gated (**e**). Ly6C^hi^ monocytes were grouped, then subgrouped for p-AKT1^Ser473+^ monocytes (**f**). (**g**) Percentage of BAL monocytes. (**h**) Percentage of p-AKT1^Ser473+^ monocytes was presented. *N*=4 in each group, *P<0.05, **P<0.01. (**i**–**l**) Flow cytometry analysis of splenic ACh-producing CD4^+^CHAT^+^cells during *E. coli* pneumonia. Using the same experimental setting as (**a**–**d**), the four groups of spleen cells were subjected to flow cytometric analysis. (**i**) Lymphocyte population was gated. (**j**) CD4^+^CHAT^+^ cells were subgated. (**k**) Percentage of splenic CD4^+^CHAT^+^ cells. (**l**) Number of CD4^+^CHAT^+^ cells was calculated by multiplying the percentage with total spleen cell counts. *N*=4 in each group, *P<0.05. Data are presented as mean±s.d.

Because vagal signals stabilize α7 nAChR^+^CD11b^+^ cells in the spleen via phosphorylation of AKT1, we tested whether vagotomy or double deletion of *Chrna7* and *Itgam* could reduce p-AKT1^ser473+^ in splenic ly6C^int^Gr1^hi^ neutrophils and ly6C^hi^ monocytes. We IT challenged sham, vagotomized and *Chrna7*^−/−^*Itgam*^−/−^ mice with *E. coli*. At 24 h, spleen cells were isolated and subjected to flow cytometry. Cells were gated (Figure 7a and e) and ly6C^int^Gr1^hi^ neutrophils and ly6C^hi^ monocytes were subgated (Figure 7b and f) to analyze percentage of p-AKT1^ser473+^ cells. The percentage of ly6C^int^Gr1^hi^ neutrophils and ly6C^hi^ monocytes in *E. coli*-infected vagotomized and *Chrna7*^−/−^*Itgam*^−/−^ mice was reduced compared to *E. coli*-infected sham mice (Figure 7c and g). Also, the percentage of p-AKT1^ser473+^ population in the ly6C^int^Gr1^hi^ neutrophils and ly6C^hi^ monocytes was decreased in *E. coli*-infected vagotomized and *Chrna7*^−/−^*Itgam*^−/−^ mice compared to *E. coli*-infected sham mice (Figure 7d and h). These findings indicate that vagal signals reduce spleen egress of neutrophils and monocytes via serine phosphorylation of AKT1. Considering that CD4^+^CHAT^+^ ACh-producing cells relay vagal outflow at spleen nerve terminus [18], we repeated experiment as Figure 7a–d at 24 h. Lymphocytes in splenic cells were gated (Figure 7i) and CD4^+^CHAT^+^ cells were subgated in normal, *E. coli*-infected sham, vagotomized, and *Chrna7*^−/−^*Itgam*^−/−^ groups (Figure 7j). The percentage and number of splenic CD4^+^CHAT^+^ cells in *E. coli*-infected vagotomized and *Chrna7*^−/−^*Itgam*^−/−^ mice were significantly reduced compared to *E. coli*-infected sham mice (Figure 7k and l). The percentage of splenic CD4^+^ cells in *E. coli*-infected vagotomized and *Chrna7*^−/−^*Itgam*^−/−^ mice were decreased relative to *E. coli*-infected wildtype mice (Supplementary Figure S10A and B). Western blotting showed that splenic p-AKT1 and CHAT expression was reduced in *Chrna7*^−/−^*Itgam*^−/−^ mice compared with *E. coli*-infected wildtype mice (Supplementary Figure S10C and D). These findings suggest that the reduction of CD4^+^CHAT^+^ cells might contribute to less serine473 phosphorylation of AKT1 in the vagotomized and *Chrna7*^−/−^*Itgam*^−/−^ mice.

### Defect of Serine473 phosphorylation of AKT1 in BAL neutrophils and monocytes from *E. coli*-infected vagotomized or Chrna7^−/−^Itgam^−/−^ mice

**Figure 8:**
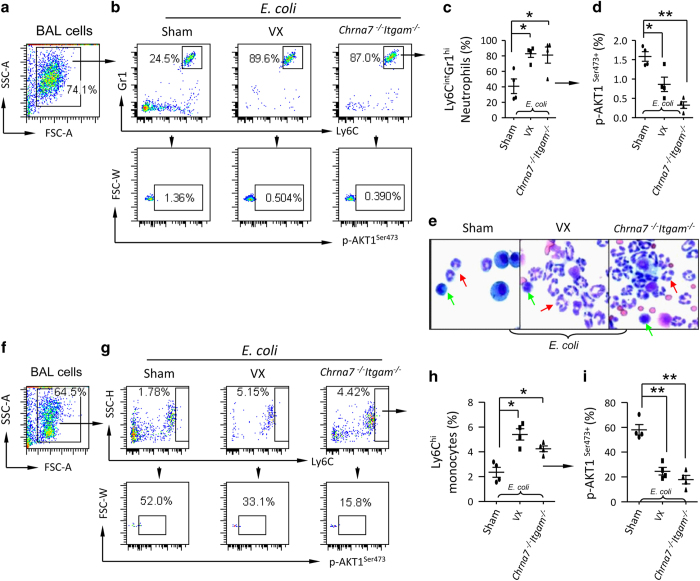
Vagotomy and double deletion of Chrna7 and Itgam reduce AKT1^Ser473^ phosphorylation in BAL neutrophils and monocytes during *E. coli *pneumonia. The sham, vagotomized and Chrna7^−/−^Igtam^−/−^ mice were IT challenged with *E. coli* (2.5×10^6^ cfu). The mice were killed at 24 h after *E. coli* challenge. BAL cells were collected and labeled with Fluorescent antibodies. (**a**–**d**) Flow cytometry analysis of AKT1^Ser473^phosphorylation in BAL neutrophils. (**a**) Whole-cell population was gated. (**b**) Ly6C^int^Gr1^hi^ neutrophils were estimated in each group. (**c**) Percentage of BAL neutrophils. (**d**) Percentage of p-AKT1^Ser473+^ neutrophils was calculated. *N*=4 in each group, *P<0.05, **P<0.01. Data are presented as mean±s.d. (**e**) Representative micrograph of Hema3 staining of BAL cells collected from* E. coli*-infected sham, vagotomized and Chrna7^−/−^Igtam^−/−^ mice. Red arrows indicate neutrophils; green arrows denote monocytes. (**f**–**i**) Flow cytometry analysis of AKT1^Ser473^phosphorylation in BAL monocytes. (**f**) Whole-cell population was gated. (**g**) Ly6C^hi^ monocytes were subgated for p-AKT1^Ser473+^ monocytes. (**h**). Percentage of BAL monocytes. (**i**) Percentage of p-AKT1^Ser473+^ monocytes was presented. *N*=4 in each group, *P<0.05, **P<0.01. Data are presented as mean±s.d.

To test whether vagotomy or double deletion of *Chrna7* and *Itgam* impaired serine473 phosphorylation of AKT1 in recruited neutrophils and monocytes during *E. coli* infection, we IT challenged sham, vagotomized and *Chrna7*^−/−^*Itgam*^−/−^ mice with *E. coli*. At 24 h, BAL cells were isolated and subjected to flow cytometry. To analyze serine473 phosphorylation of AKT1 in BAL neutrophils, we first gated the whole-cell population (Figure 8a) and subgated the ly6C^int^Gr1^hi^ neutrophils in each group (Figure 8b). The percentage of p-AKT1^ser473+^ population was calculated from ly6C^int^Gr1^hi^ cells. We found that the percentage of ly6C^int^Gr1^hi^ neutrophils in BAL from *E. coli*-infected vagotomized or *Chrna7*^−/−^*Itgam*^−/−^ mice was increased compared to *E. coli*-infected sham mice (Figure 8c). The percentage of p-AKT1^ser473+^ population in ly6C^int^Gr1^hi^ neutrophils in BAL from *E. coli*-infected vagotomized or *Chrna7*^−/−^*Itgam*^−/−^ mice was decreased compared to *E. coli*-infected sham mice (Figure 8d). The histology of isolated BAL cells also showed the number of neutrophils was markedly higher in the *E. coli*-infected vagotomized or *Chrna7*^−/−^*Itgam*^−/−^ mice (Figure 8e). Using the same gating strategies (Figure 8f and g), we found that the percentage of ly6C^hi^ monocytes in BAL from *E. coli*-infected vagotomized or *Chrna7*^−/−^*Itgam*^−/−^ mice was increased compared to *E. coli*-infected sham mice (Figure 8h). The percentage of p-AKT1^ser473+^ population in ly6C^hi^ monocytes in BAL from *E. coli*-infected vagotomized or *Chrna7*^−/−^*Itgam*^−/−^ mice was decreased compared to *E. coli*-infected sham mice (Figure 8i). We used the same experimental setting and found that lung *Il22* mRNA was reduced and *Cxcl2* mRNA was increased in *E. coli*-infected vagotomized or *Chrna7*^−/−^*Itgam*^−/−^ mice (Supplementary Figure S11A and B). BM monocytes (Ly6C^hi^Ly6G^int^) in *E. coli*-infected vagotomized and *Chrna7*^*−/−*^*Itgam*^*−/−*^ mice were increased compared to *E.coli*-infected wildtype mice, whereas BM neutrophils (Ly6C^int^Ly6G^*hi*^) were not different among these three groups (Supplementary Figure S11C–E). These findings indicate that vagal signals regulate splenic neutrophils and monocytes to the infected lungs via serine phosphorylation of AKT1.

### Deletion of Itgam or/and Chrna7 worsens *E. coli* and LPS-induced ALI

**Figure 9:**
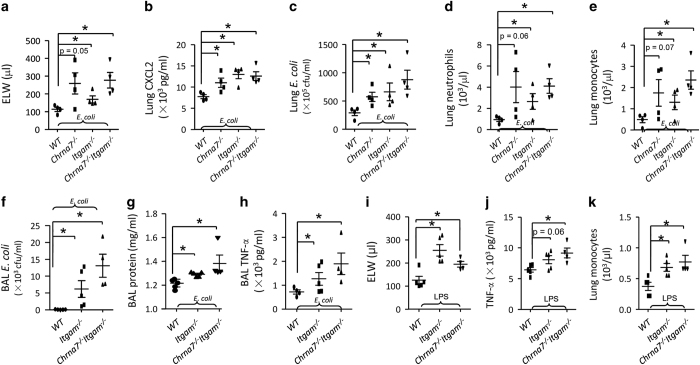
Deletion of *Itgam* or/and *Chrna7* worsens *E. coli* and LPS-induced ALI. Double deletion of *Chrna7* and *Itgam* aggravates *E. coli*-induced ALI (**a**–**e**). The wildtype, *Chrna7*^−/−^, *Itgam*^−/−^ and *Chrna7*^−/−^*Itgram*^−/−^ mice were, respectively, challenged with *E. coli* (2.5×10^6^ cfu) intratracheally. The mice were killed at 24 h after *E. coli* challenge. ELW (**a**), CXCL2 levels (**b**) in the supernatant of lung homogenate, lung *E. coli* colonies (**c**), lung neutrophils (**d**) and lung monocytes (**e**) were measured. *N*=4 in each group, *P<0.05, one-way ANOVA with Bonferroni *post hoc* test. Data are presented as mean±s.d. Double deletion of *Chrna7* and *Itgam* increases BAL inflammatory parameters in *E. coli*-induced ALI (**f**–**h**). The wildtype, *Itgam*^−/−^ and *Chrna7*^−/−^Itgram^−/−^ mice were, respectively, challenged with *E. coli* (2.5×10^6^ cfu) intratracheally. The mice were killed at 24 h after *E. coli* challenge. BAL was collected to measure BAL *E. coli *colonies (**f**), BAL protein (**g**) and BAL TNF-α (**h**). *N*=4 in each group, *P<0.05, one-way ANOVA with Bonferroni *post hoc* test. Data are presented as mean±s.d. (**i**–**k**) Double deletion of *Chrna7* and *Itgam* exacerbates LPS-induced ALI. The wildtype, *Itgam*^−/−^ and *Chrna7*^−/−^*Itgam*^−/−^ mice were, respectively, challenged with LPS (5 mg kg^−1^) intratracheally. The mice were killed at 24 h after LPS challenge. ELW (**i**), TNF-α level (**j**) in the supernatant of lung homogenate and lung monocytes (**k**) were analyzed. *N*=5 in each group, *P<0.05, one-way ANOVA with Bonferroni *post hoc* test. Data are presented as mean±s.d.

Peritoneal macrophages were isolated from wildtype, *Chrna7*^*−/−*^, *Itgam*^*−/−*^ and *Chrna7*^*−/−*^*Itgam*^*−/−*^ mice, and stimulated with LPS for 4 h. TNF-α in the supernatant of culture media was significantly increased in LPS-challenged *Chrna7*^*−/−*^, *Itgam*^*−/−*^ and *Chrna7*^*−/−*^*Itgam*^*−/−*^ macrophages (Supplementary Figure S12). We tested whether deletion of *Itgam or/and Chrna7* would worsen *E. coli* pneumonia. To this end, we IT challenged mice with WT, *Chrna7*^*−/−*^, *Itgam*^*−/−*^ and *Chrna7*^*−/−*^*Itgam*^*−/−*^ mice with *E. coli*. We found that ELW (Figure 9a), CXCL2 (Figure 9b), *E. coli* colonies (Figure 9c), neutrophils and monocytes (Figure 9d and e) in the lungs were increased in *E. coli-*infected *Chrna7*^*−/−*^, *Itgam*^*−/−*^ and *Chrna7*^*−/−*^*Itgam*^*−/−*^ groups compared with *E. coli-*infected WT group. We also collected BAL in *E. coli* pneumonia and found that BAL *E. coli* colonies (Figure 9f), protein (Figure 9g) and TNF-α level (Figure 9h) were increased in *E. coli-*infected *Itgam*^*−/−*^and *Chrna7*^*−/−*^*Itgam*^*−/−*^ groups compared with *E. coli-*infected WT group.

To study whether deletion of *Itgam* or/and *Chrna7* would exacerbate LPS-induced ALI, we IT challenged wildtype, *Itgam*^*−/−*^ and *Chrna7*^*−/−*^*Itgam*^*−/−*^ mice with LPS. There were more severe pulmonary edema (Figure 9i) and higher lung TNF-α (Figure 9j) and monocytes (Figure 9k) in LPS-challenged *Itgam*^*−/−*^ and *Chrna7*^*−/−*^*Itgam*^*−/−*^ mice compared with the LPS-challenged WT group; however, these parameters were not different between the LPS-challenged *Itgam*^*−/−*^ group and *Chrna7*^*−/−*^*Itgam*^*−/−*^ group (Figure 9i–k). These findings support that α7 nAChR^+^CD11b^+^ cells have a protective role during *E. coli* and LPS-induced lung injury.

## Discussion

α7 nAChR-expressing macrophages and CD4^+^CHAT^+^ cells in the spleen are the effector cells for CAP to downregulate systemic inflammatory responses [18]. To elucidate the role of signals of vagal circuits in regulating local (lung) inflammatory responses, we postulated a pulmonary parasympathetic inflammatory reflex [30, 31] in which α7 nAChR-expressing CD11b^+^ and Gr1^+^ cells are efferent arms of this reflex. Activation of α7 nAChR with ACh (neurotransmitter of vagal circuits) in CD11b^+^ cells could dampen *E. coli* and LPS-induced inflammatory responses [30, 31]. Disruption of vagal circuits inactivated α7 nAChR in CD11b^+^ cells and reduced *E. coli* and LPS-induced inflammatory responses.

Our data showed that more CD11b^+^ granulocytes were discharged from spleen and recruited to the lung in *E. coli-*infected *Chrna7*^−/−^ mice. The *E. coli-*infected *Akt1*^−/−^ mice shared the similar phenotype as *Chrna7*^−/−^ mice, suggesting that AKT1 has a role in regulating flux and function of α7 nAChR-expressing CD11b^+^ cells. CD11b is a negative regulator for TLR4 signaling and CD11b-deficienct mice were susceptible to bacterial infection [41]. There were more CD11b^+^ cells presented in *E. coli-*infected *Chrna7*^−/−^ or *Akt1*^−/−^ lung, but inflammation was worsened, suggesting that CD11b is not able to exert inhibitory effect on inflammation without function of α7 nAChR and AKT1.

On the basis of the results of these experiments, did the spleen alone account for the accumulation of CD11b^+^ cells in the lungs? Considering that mobilization of bone marrow proinflammatory cells is an important pathological process in response to lung infection [46], we had to clarify whether bone marrow was a source of inflammatory cells under *E. coli*-infected vagotomized and *Chrna7*^*−/−*^*Itgam*^*−/−*^ conditions. By flow cytometry, we found that changes of monocytes and neutrophils in the bone marrow were not in accordance with changes of accumulated lung proinflammatory cells. Furthermore, peritoneal macrophages are resident cells and these cells do not migrate to the lung during lung infection and inflammation. Therefore, our findings strongly suggest that lung inflammatory cells are most likely recruited from the spleen under *E.coli*-infected vagotomized and *Chrna7*^*−/−*^*Itgam*^*−/−*^ conditions.

Another important question is why neutrophils and monocytes accumulated in the vagotomized and *Chrna7*^*−/−*^*Itgam*^*−/−*^ lungs but did not reduce bacterial infection. The possible explanation is that acetylcholine or α7 nAChR agonist could modify their anti-bacterial properties. Sitapara *et al.* [47] reported that activation of α7 nAChR by GTS-21 is effective in improving bacterial clearance and decreasing acute lung injury. GTS-21 can improve the phagocytic ability of hyperoxic macrophages. It was found that cholinergic stimulation enhanced the antimicrobial immune response leading to effective control of bacterial proliferation and improved survival [48]. ChAT-expressing T cells were found to control the host antimicrobial peptide secretion, microbial growth and expansion [49]. Serhan’s group reported that *E. coli* phagocytosis was impaired in peritoneal exudate Ly6G^+^CD11b^+^ neutrophils in right cervical vagotomized mice [50]. They found that peritoneal acetylcholine levels were significantly reduced d1, 3 and 7 post right cervical vagotomy [50]. Right rather than left vagus nerve regulates host responses to bacterial infection [50]. Dissection of the right vagus decreased peritoneal group 3 innate lymphoid cell (ILC3) numbers and altered peritoneal macrophage responses to bacteria infection. Stimulation of acetylcholine in ILC3s could enhance yield of immunoresolvent PCTR1, which protected bacterial infection [50]. ILC3 is the major source of IL-22, and the later is a protective mediator of mucosa immunity [51, 52]. In this study, lung *Il22* mRNA was reduced and *Cxcl2* mRNA was increased in *E. coli*-challenged vagotomized and *Chrna7*^*−/−*^*Itgam*^*−/−*^ mice compared to the control. The findings suggested that vagotomy and deficiency of α7 nAChR and CD11b compromise anti-bacterial capacity and promote proinflammatory cytokine production.

A third issue is how AKT1 regulates migration of proinflammatory cells. It has been reported that AKT1-deficient neutrophils exhibit an enhanced migration and production of proinflammatory cytokines [43]. AKT1 has a negative role in regulating migration of neutrophils and development of acute inflammation [53–55]. In this study, the p-AKT1^Ser473^ levels were markedly decreased in the isolated neutrophils from LPS-challenged vagotomized mice. Activation of α7 nAChR promoted phosphorylation of AKT1^Ser473^ and inhibited CXCL2 production. Inhibition of phosphorylation of AKT1^Ser473^ enhanced CXCL2 production in neutrophils. Vagotomy markedly decreased phosphorylation of AKT1^Ser473^ in splenic and BAL α7 nAChR^+^CD11b^+^ cells, or Ly6C^int^Gr1^hi^ neutrophils and Ly6C^hi^ monocytes, facilitated alveolar recruitment of inflammatory cells, and deteriorated lung inflammation. In addition, phosphorylation of Bax Ser184 by AKT regulates its activity and apoptosis in neutrophils [56]. Initiation of the apoptotic process inhibits the ability of neutrophils to move, degranulate, and produce superoxide [57]. AKT1 might be essential to induce NADPH-dependent NETosis and to switch the neutrophil death to apoptosis [58]. Inflammatory mediators could inhibit apoptosis of neutrophils and prolong neutrophil functional longevity [57]. Therefore, we speculate that AKT1 might regulate migration of neutrophils via impacting on apoptosis, which is worthy of further investigation in the future.

Another issue is to determine the fate of CD4^+^CHAT^+^ lymphocytes in the spleen. Spleen CD4^+^CHAT^+^ lymphocytes synthesize acetylcholine and render anti-inflammatory activity to splenic Ly6C^int^Gr1^hi^ neutrophils and Ly6C^hi^ monocytes via phosphorylation of AKT1. Vagotomy and double deletion of *Chrna7* and *Itgam* reduced spleen CD4^+^CHAT^+^ lymphocytes. The percentage of CD4^+^ T cells was reduced, suggesting that CD4^+^ T cells might egress from vagotomized spleens or undergo apoptotic. However, this finding is not consistent with the previous report that vagotomy facilitated proliferation of CD4^+^ T cells [59]. The spleen receives innervation of both sympathetic and parasympathetic nerves [60, 61]. Activation of sympathetic β2-adrenergic receptor promotes CHAT expression in the spleen. We found that CHAT expression was reduced in *Chrna7*^*−/−*^*Itgam*^*−/−*^ spleens. This finding suggests that α7 nAChR or/and CD11 also regulate CHAT expression as a feedback.

The anti-inflammatory role of CAP in the spleen was confirmed by splenectomy, [18, 23, 24] as splenectomy could inactivate CAP during lethal endotoxemia and polymicrobial sepsis [23]. However, some studies have demonstrated that splenectomy protected against sepsis lethality and reduced serum HMGB1 levels [62], and splenectomy was detrimental to the immune response to lung infection [63]. Splenectomy causes immunologic impairment and increases susceptibility to infection [64–66]. Therefore, splenectomy might confound the findings of *E. coli* lung infection.

We aimed to observe acute effect of vagotomy on lung infection and inflammation, so we chose challenging the mice immediately after vagotomy. However, a study suggested that surgical vagotomy reduced plasma TNF-α production probably because of nerve damage-triggered ACh release [67], but blood ACh levels were not measured in that study. In this study, α7 nAChR agonist could reverse LPS and *E. coli*-induced lung proinflammatory responses deteriorated by vagotomy, and this result does not support that vagotomy triggers release of ACh. Pulmonary neurectomy might be an alternative way to test specificity of vagal denervation on lung recruitment of α7 nAChR^+^CD11b^+^ cells and development of inflammation. However, the pulmonary branches of vagus nerve are joined by filaments from sympathetic nerve; it is impractical to selectively disrupt lung vagal innervation.

In summary, during *E. coli* and LPS-induced ALI, splenic CD4^+^CHAT^+^ cells can synthesize ACh, which activates α7 nAChR in CD11b^+^ cells (monocytes and neutrophils) to elicit phosphorylation of AKT1, stabilizes α7 nAChR^+^CD11b^+^ cells in the spleen and decreases recruitment of α7 nAChR^+^CD11b^+^ cells towards the injured lung. In addition, vagal efferent nerve endings innervate distal lung parenchyma or alveoli where ACh is released upon infection or LPS stimulation. Activation of α7 nAChR by ACh in the recruited CD11b^+^ cells could mitigate production of proinflammatory cytokines and bacterial growth and therefore attenuate lung inflammation and infection. Disruption of vagal circuits reduces synthesis of ACh in CD4^+^CHAT^+^ cells and impairs phosphorylation of AKT1 of α7 nAChR^+^CD11b^+^ cells in the spleen. This event facilitates splenic egress and lung recruitment of α7 nAChR^+^CD11b^+^ cells. Disruption of intact vagal circuits or lack of sufficient ACh impairs phosphorylation of AKT1 in the recruited α7 nAChR^+^CD11b^+^ cells. The proinflammatory responses in these cells could not be efficiently attenuated. Similar to vagotomy, double deletion of *Chrna7* and *Itgam* reduces splenic CD4^+^CHAT^+^ cells and phosphorylation of AKT1 in neutrophils and monocytes, and promote recruitment of these cells to the lung where lung proinflammatory responses are propagated (Supplementary Figure S13). Thus, signals of vagal circuits engaging with phosphorylation of AKT1 in α7 nAChR^+^CD11b^+^ cells attenuate *E. coli* and LPS-induced acute lung inflammatory responses.

## Materials and Methods

### Animals

*Chrna7*^−/−^, B6.129S7-*Chrna7*^*tm1Bay*^*/J; Akt1*^−/−^, B6.129P2-*Akt1*^*tm1Mbb*^*/J; Itgam*^−/−^, B6.129S4-*Itgam*^*tm1Myd*^*/J; Tlr4*^−/−^, B6.B10ScN-*Tlr4*^*lps-del*^*/JthJ* and wildtype mice (C57BL/6) were purchased from the Jackson Laboratory (Bar Harbor, ME, USA) and the Model Animal Research Center of Nanjing University (Nanjing, China). LysM-EGFP was from E. Robey (UC, Berkeley, CA, USA) [45]. The mice were housed with 12 h dark/light cycles and with free access to food and water. Anesthesia was induced with an intraperitoneal (ip) injection of a mixture of xylazine (10 mg kg^−1^) and ketamine (90 mg kg^−1^) or pentobarbital sodium (50 mg kg^−1^). The Committees on Animal Research of the Institut Pasteur of Shanghai, Chinese Academy of Sciences approved the animal studies.

### Mouse animal models

Procedures to establish LPS-induced ALI and *E. coli* pneumonia have been published in our previous studies [28, 29]. There was no death in both control and treated groups if receiving an IT *E. coli* (2.5×10^6^ cfu) at 24 h [29].

### Unilateral vagotomy

Right or sham cervical vagotomy was performed with the animals under anesthesia. The procedure involved a longitudinal midline incision in the ventral region of the neck. Using blunt dissection, the overlying muscles and fascia were separated until the right vagus and carotid artery were visible. The vagus was carefully stripped away from carotid artery and lightly cut off in the vagotomy group. The vagus was kept intact in sham group. The wound was closed and sutured. The respiration rhythm was not affected by unilateral vagotomy. It is reported that right nerve controls cardiac function and this side is not chosen for vagus nerve stimulation [68]. In our study, comparing sham to vagotomy mice receiving an IT of saline, there no any difference in BAL cytokines and inflammatory cell profiles within 24 h. No mice died of heart failure after right side of vagotomy.

### Reagents

DMAB-anabaseine dihydrochloride and PHA568487 were from Tocris Biosciences (Minneapolis, MN, USA); (−)-Nicotine hemisulfate salt and LPS *E. coli* 0111:B4 were from Sigma (St Louis, MO, USA). Anti-mouse Ly6C APC, anti-mouse CD11b PE, anti-mouse Gr1 FITC and PE, anti-mouse CD4 FITC and PE, anti-mouse CD16/CD32 antibodies and IgG isotype controls were from eBioscience (San Diego, CA, USA). For immunofluorescence, anti-mouse CD11b and Gr1 antibodies were from BD Biosciences (San Jose, CA, USA). Phospho-Akt1 (Ser473) (D7F10) XP rabbit mAb (Akt1 Specific) and PathScan Phospho-Akt1 (Ser473) Sandwich ELISA Kit were from Cell Signaling (Danvers, MA, USA); AChRα7 antibody (H-302) was from Santa Cruz Biotechnology (Santa Cruz, CA, USA). Mouse CCR2 APC-conjugated antibody and mouse CXCL2 and TNF-α ELISA Kit were from R&D Systems (Minneapolis, MN, USA). Anti-Choline acetyltransferase antibody was from Abcam (Cambridge, MA, USA). *E. coli* K1 (serotype) strain, isolated from patients with biliary infection, was kindly provided by Dr Thomas Martin (University of Washington, USA) [69].

### Measurement of ELW

The detailed procedures were described previously [28]. Briefly, homogenate and supernatant of lung, and blood were weighed and then desiccated in an oven (60 °C for 24 h). ELW was calculated by standard formula:

$$\mathrm{ELW}=\left[\left(Q_{W,\;\;\exp}/Q_{d,\;\;\exp}\times Q_{d,\;\;\exp}\right)-\left(Q_{W,\;\;\mathrm{control}}{/Q}_{d,\;\;\mathrm{control}}\times Q_{d,\;\;\mathrm{control}}\right)\right]\times 1\;000(µl)$$

where *Q*_W,exp_ equals water volume of the lung in the experimental group; *Q*_d,exp_ equals dry weight of lung in the experimental group. The controls were the normal mice with the same age as the experimental group.

### Immunofluorescence and determination of lung myeloperoxidase activity

Briefly, we performed Immunofluorescence referring to the previous study [29]. To measure lung myeloperoxidase (MPO) activity, the supernatants of lung homogenate were mixed with *o*-dianisidine HCl (Sigma-Aldrich) and H_2_O_2_ to measure optical density by a spectrophotometer at 405 nm using our established method [29].

### Bronchoalveolar lavage, protein levels and leukocyte counts and differentiation

The detailed procedures were described previously [28, 29]. The mice were intubated with a 24-gauge cannula after their tracheas were surgically isolated. The lungs were flushed with 1 ml PBS back and forth for 3 times. Protein concentration in the BAL was determined by a Bio-Rad protein assay (Bio-Rad Laboratories, Hercules, CA, USA). BAL cell smear was made using cytospin (Shandon, Pittsburgh, PA, USA). The slides were visualized using Hema3 staining (Fisher Scientific, Middletown, VA, USA). Neutrophils and monocytes were identified by a certified laboratory technologist in a blinded fashion. BAL cells were also used for flow cytometry analysis after lysing erythrocytes.

### Complete blood count

Using a multispecies hematology instrument (Hemavet 950FS; Drew Scientific, Dallas, TX, USA), we measured blood neutrophils, monocytes, hematocrit and other parameters [40].

### Preparation of lung cells, spleen and blood cells

Harvested lungs were inflated with 3 ml of a mixture of collagenase (150 U ml^−1^) and DNaseI (10 μg ml^−1^) in RPMI-1640 containing 5% FBS and 20 mm HEPES. Lungs were chopped in the 3 ml enzyme mix and incubated for 35 min at 37 °C. Overall, 10 mm EDTA was added and any remaining pieces were further dispersed by 12 passages through a 21-G needle. Suspensions were passed through a 100 μm nylon mesh and cells were washed multiple times in RPMI-1640 with 5% FBS, 20 mm HEPES. RBC lysis was performed on cell preparations for cellular analysis by flow cytometry. For preparing single cells, spleens were removed and grounded in a 70 μm cell strainer. Erythrocytes were lysed [11]. For preparing blood leukocytes, we utilized BD Pharm Lyse-Lysing Buffer (BD Biosciences) to lyse erythrocytes.

### Flow cytometric analysis

After unspecific staining was minimized through pre-incubation for 15 min with anti-mouse CD16/32 antibodies, lung, spleen, blood or BAL cells were labeled with primary or isotype antibodies. Isotype antibody and unstained controls were used to demonstrate specificity of staining and to establish the criteria for flow cytometry populations (for simplicity, data are not presented regarding these controls). The data fluorescent cells were analyzed after excluding debris and aggregates with LSRFortessa (BD Biosciences). Data were analyzed by *Flowjo* 7.6 software (Tree Star, Ashland, OR, USA).

### Measurements of cytokine levels by ELISA

TNF-α, CXCL2, and phospho-Akt1 (Ser473) levels in the biofluid were measured by ELISA kits according to the manufacturers’ instructions.

### Statistical analysis

Statistics analysis was performed using GraphPad Prism software (GraphPad, San Diego, CA, USA). For multiple comparisons, we chose one-way ANOVA with Bonferroni *post hoc* test. For comparing two groups, we approached a Student’s *t*-test. For repeated measures, we used two-way ANOVA. We examined difference of survival by a log-rank test. Significance level was set at *P*<0.05. The results are shown as mean±s.d.

## References

[bib1] Ware LB, Matthay MA. The acute respiratory distress syndrome. N Engl J Med 2000; 342: 1334–1349. 10793167 10.1056/NEJM200005043421806

[bib2] Wiener-Kronish JP, Gropper MA, Matthay MA. The adult respiratory distress syndrome: definition and prognosis, pathogenesis and treatment. Br J Anaesth 1990; 65: 107–129. 2200479 10.1093/bja/65.1.107

[bib3] Pittet JF, Mackersie RC, Martin TR, Matthay MA. Biological markers of acute lung injury: prognostic and pathogenetic significance. Am J Respir Crit Care Med 1997; 155: 1187–1205. 9105054 10.1164/ajrccm.155.4.9105054

[bib4] Kaslovsky RA, Parker K, Siflinger-Birnboim A, Malik AB. Increased endothelial permeability after neutrophil activation occurs by a diffusion-dependent mechanism. Microvasc Res 1995; 49: 227–232. 7603357 10.1006/mvre.1995.1018

[bib5] Gardinali M, Borrelli E, Chiara O et al. Inhibition of CD11-CD18 complex prevents acute lung injury and reduces mortality after peritonitis in rabbits. Am J Respir Crit Care Med 2000; 161: 1022–1029. 10712358 10.1164/ajrccm.161.3.9901066

[bib6] Zhou MY, Lo SK, Bergenfeldt M et al. *In vivo* expression of neutrophil inhibitory factor via gene transfer prevents lipopolysaccharide-induced lung neutrophil infiltration and injury by a beta2 integrin-dependent mechanism. J Clin Invest 1998; 101: 2427–2437. 9616214 10.1172/JCI407 PMC508832

[bib7] Su X. Leading neutrophils to the alveoli: who is the guider? Am J Respir Crit Care Med 2012; 186: 472–473. 22984021 10.1164/rccm.201207-1235ED

[bib8] Dhaliwal K, Scholefield E, Ferenbach D et al. Monocytes control second-phase neutrophil emigration in established lipopolysaccharide-induced murine lung injury. Am J Respir Crit Care Med 2012; 186: 514–524. 22822022 10.1164/rccm.201112-2132OC PMC3480527

[bib9] Tracey KJ. The inflammatory reflex. Nature 2002; 420: 853–859. 12490958 10.1038/nature01321

[bib10] Andersson J. The inflammatory reflex—introduction. J Intern Med 2005; 257: 122–125. 15656871 10.1111/j.1365-2796.2004.01440.x

[bib11] Gabanyi I, Muller PA, Feighery L, Oliveira TY, Costa-Pinto FA, Mucida D. Neuro-immune interactions drive tissue programming in intestinal macrophages. Cell 2016; 164: 378–391. 26777404 10.1016/j.cell.2015.12.023 PMC4733406

[bib12] Andersson U, Tracey KJ. Neural reflexes in inflammation and immunity. J Exp Med 2012; 209: 1057–1068. 22665702 10.1084/jem.20120571 PMC3371736

[bib13] Ogbonnaya S, Kaliaperumal C. Vagal nerve stimulator: evolving trends. J Nat Sci Biol Med 2013; 4: 8–13. 23633829 10.4103/0976-9668.107254 PMC3633308

[bib14] Pavlov VA, Tracey KJ. Neural regulators of innate immune responses and inflammation. Cell Mol Life Sci 2004; 61: 2322–2331. 15378203 10.1007/s00018-004-4102-3 PMC11138906

[bib15] Gallowitsch-Puerta M, Pavlov VA. Neuro-immune interactions via the cholinergic anti-inflammatory pathway. Life Sci 2007; 80: 2325–2329. 17289087 10.1016/j.lfs.2007.01.002 PMC2921074

[bib16] Tracey KJ. Reflex control of immunity. Nat Rev Immunol 2009; 9: 418–428. 19461672 10.1038/nri2566 PMC4535331

[bib17] Tracey KJ. Reflexes in Immunity. Cell 2016; 164: 343–344. 26824649 10.1016/j.cell.2016.01.018

[bib18] Rosas-Ballina M, Olofsson PS, Ochani M et al. Acetylcholine-synthesizing T cells relay neural signals in a vagus nerve circuit. Science 2011; 334: 98–101. 21921156 10.1126/science.1209985 PMC4548937

[bib19] Andersson U, Tracey KJ. Reflex principles of immunological homeostasis. Ann Rev Immunol 2012; 30: 313–335. 22224768 10.1146/annurev-immunol-020711-075015 PMC4533843

[bib20] Rosas-Ballina M, Tracey KJ. The neurology of the immune system: neural reflexes regulate immunity. Neuron 2009; 64: 28–32. 19840545 10.1016/j.neuron.2009.09.039 PMC4533851

[bib21] Wang H, Liao H, Ochani M et al. Cholinergic agonists inhibit HMGB1 release and improve survival in experimental sepsis. Nat Med 2004; 10: 1216–1221. 15502843 10.1038/nm1124

[bib22] Wang H, Yu M, Ochani M et al. Nicotinic acetylcholine receptor alpha7 subunit is an essential regulator of inflammation. Nature 2003; 421: 384–388. 12508119 10.1038/nature01339

[bib23] Huston JM, Ochani M, Rosas-Ballina M et al. Splenectomy inactivates the cholinergic antiinflammatory pathway during lethal endotoxemia and polymicrobial sepsis. J Exp Med 2006; 203: 1623–1628. 16785311 10.1084/jem.20052362 PMC2118357

[bib24] Rosas-Ballina M, Ochani M, Parrish WR et al. Splenic nerve is required for cholinergic antiinflammatory pathway control of TNF in endotoxemia. Proc Natl Acad Sci USA 2008; 105: 11008–11013. 18669662 10.1073/pnas.0803237105 PMC2504833

[bib25] Fox B, Bull TB, Guz A. Innervation of alveolar walls in the human lung: an electron microscopic study. J Anat 1980; 131: 683–692. 7216905 PMC1233220

[bib26] Hertweck MS, Hung KS. Ultrastructural evidence for the innervation of human pulmonary alveoli. Experientia 1980; 36: 112–113. 7358113 10.1007/BF02004006

[bib27] Livermore S, Zhou Y, Pan J, Yeger H, Nurse CA, Cutz E. Pulmonary neuroepithelial bodies are polymodal airway sensors: evidence for CO_2_/H^+^ sensing. Am J Physiol Lung Cell Mol Physiol 2015; 308: L807–L815. 25659901 10.1152/ajplung.00208.2014

[bib28] Su X, Lee JW, Matthay ZA et al. Activation of the alpha7 nAChR reduces acid-induced acute lung injury in mice and rats. Am J Respir Cell Mol Biol 2007; 37: 186–192. 17431097 10.1165/rcmb.2006-0240OC PMC1976545

[bib29] Su X, Matthay MA, Malik AB. Requisite role of the cholinergic alpha7 nicotinic acetylcholine receptor pathway in suppressing Gram-negative sepsis-induced acute lung inflammatory injury. J Immunol 2010; 184: 401–410. 19949071 10.4049/jimmunol.0901808 PMC2877486

[bib30] Yang X, Zhao C, Gao Z, Su X. A novel regulator of lung inflammation and immunity: pulmonary parasympathetic inflammatory reflex. QJM 2014; 107: 789–792. 24440925 10.1093/qjmed/hcu005

[bib31] Wu H, Li L, Su X. Vagus nerve through alpha7 nAChR modulates lung infection and inflammation: models, cells, and signals. Biomed Res Int 2014; 2014: 283525. 25136575 10.1155/2014/283525 PMC4127262

[bib32] Huston JM, Rosas-Ballina M, Xue X et al. Cholinergic neural signals to the spleen down-regulate leukocyte trafficking via CD11b. J Immunol 2009; 183: 552–559. 19542466 10.4049/jimmunol.0802684 PMC2806576

[bib33] Davis HM, Carpenter DC, Stahl JM, Zhang W, Hynicka WP, Griswold DE. Human granulocyte CD11b expression as a pharmacodynamic biomarker of inflammation. J Immunol Methods 2000; 240: 125–132. 10854607 10.1016/s0022-1759(00)00183-6

[bib34] Zhou X, Gao XP, Fan J et al. LPS activation of Toll-like receptor 4 signals CD11b/CD18 expression in neutrophils. Am J Physiol Lung Cell Mol Physiol 2005; 288: L655–L662. 15563689 10.1152/ajplung.00327.2004

[bib35] Fan ST, Edgington TS. Integrin regulation of leukocyte inflammatory functions. CD11b/CD18 enhancement of the tumor necrosis factor-alpha responses of monocytes. J Immunol 1993; 150: 2972–2980. 8095957

[bib36] Powner DJ, Pettitt TR, Anderson R, Nash GB, Wakelam MJ. Stable adhesion and migration of human neutrophils requires phospholipase D-mediated activation of the integrin CD11b/CD18. Mol Immunol 2007; 44: 3211–3221. 17346796 10.1016/j.molimm.2007.01.033

[bib37] Zen K, Guo YL, Li LM, Bian Z, Zhang CY, Liu Y. Cleavage of the CD11b extracellular domain by the leukocyte serprocidins is critical for neutrophil detachment during chemotaxis. Blood 2011; 117: 4885–4894. 21403131 10.1182/blood-2010-05-287722 PMC3100697

[bib38] Overbeek SA, Kleinjan M, Henricks PA et al. Chemo-attractant N-acetyl proline-glycine-proline induces CD11b/CD18-dependent neutrophil adhesion. Biochim Biophys Acta 2013; 1830: 2188–2193. 23041749 10.1016/j.bbagen.2012.09.023

[bib39] Zhang X, Bajic G, Andersen GR, Christiansen SH, Vorup-Jensen T. The cationic peptide LL-37 binds Mac-1 (CD11b/CD18) with a low dissociation rate and promotes phagocytosis. Biochim Biophys Acta 2016; 1864: 471–478. 26876535 10.1016/j.bbapap.2016.02.013

[bib40] Wan M, van der Does AM, Tang X, Lindbom L, Agerberth B, Haeggstrom JZ. Antimicrobial peptide LL-37 promotes bacterial phagocytosis by human macrophages. J Leukoc Biol 2014; 95: 971–981. 24550523 10.1189/jlb.0513304

[bib41] Han C, Jin J, Xu S, Liu H, Li N, Cao X. Integrin CD11b negatively regulates TLR-triggered inflammatory responses by activating Syk and promoting degradation of MyD88 and TRIF via Cbl-b. Nat Immunol 2010; 11: 734–742. 20639876 10.1038/ni.1908

[bib42] Dajas-Bailador F, Wonnacott S. Nicotinic acetylcholine receptors and the regulation of neuronal signalling. Trends Pharmacol Sci 2004; 25: 317–324. 15165747 10.1016/j.tips.2004.04.006

[bib43] Liu G, Bi Y, Wang R et al. Kinase AKT1 negatively controls neutrophil recruitment and function in mice. J Immunol 2013; 191: 2680–2690. 23904165 10.4049/jimmunol.1300736

[bib44] Swirski FK, Nahrendorf M, Etzrodt M et al. Identification of splenic reservoir monocytes and their deployment to inflammatory sites. Science 2009; 325: 612–616. 19644120 10.1126/science.1175202 PMC2803111

[bib45] Looney MR, Thornton EE, Sen D, Lamm WJ, Glenny RW, Krummel MF. Stabilized imaging of immune surveillance in the mouse lung. Nat Methods 2011; 8: 91–96. 21151136 10.1038/nmeth.1543 PMC3076005

[bib46] Bajrami B, Zhu H, Kwak HJ et al. G-CSF maintains controlled neutrophil mobilization during acute inflammation by negatively regulating CXCR2 signaling. J Exp Med 2016; 213: 1999–2018. 27551153 10.1084/jem.20160393 PMC5030805

[bib47] Sitapara RA, Antoine DJ, Sharma L et al. The alpha7 nicotinic acetylcholine receptor agonist GTS-21 improves bacterial clearance in mice by restoring hyperoxia-compromised macrophage function. Mol Med 2014; 20: 238–247. 24664237 10.2119/molmed.2013.00086 PMC4069272

[bib48] Fernandez-Cabezudo MJ, Lorke DE, Azimullah S et al. Cholinergic stimulation of the immune system protects against lethal infection by *Salmonella enterica* serovar Typhimurium. Immunology 2010; 130: 388–398. 20408892 10.1111/j.1365-2567.2009.03238.x PMC2913218

[bib49] Dhawan S, De Palma G, Willemze RA et al. Acetylcholine-producing T cells in the intestine regulate antimicrobial peptide expression and microbial diversity. Am J Physiol Gastrointest Liver Physiol 2016; 311: G920–G933. 27514477 10.1152/ajpgi.00114.2016

[bib50] Dalli J, Colas RA, Arnardottir H, Serhan CN. Vagal regulation of group 3 innate lymphoid cells and the immunoresolvent PCTR1 controls infection resolution. Immunity 2017; 46: 92–105. 28065837 10.1016/j.immuni.2016.12.009 PMC5283610

[bib51] Rankin LC, Girard-Madoux MJ, Seillet C et al. Complementarity and redundancy of IL-22-producing innate lymphoid cells. Nat Immunol 2016; 17: 179–186. 26595889 10.1038/ni.3332 PMC4720992

[bib52] Van Maele L, Carnoy C, Cayet D et al. Activation of Type 3 innate lymphoid cells and interleukin 22 secretion in the lungs during *Streptococcus pneumoniae* infection. J Infect Dis 2014; 210: 493–503. 24577508 10.1093/infdis/jiu106

[bib53] Liu GW, Bi YJ, Wang RN et al. Kinase AKT1 negatively controls neutrophil recruitment and function in mice. J Immunol 2013; 191: 2680–2690. 23904165 10.4049/jimmunol.1300736

[bib54] Chen J, Tang HY, Hay N, Xu JS, Ye RD. Akt isoforms differentially regulate neutrophil functions. Blood 2010; 115: 4237–4246. 20332370 10.1182/blood-2009-11-255323 PMC2879106

[bib55] Di Lorenzo A, Fernandez-Hernando C, Cirino G, Sessa WC. Akt1 is critical for acute inflammation and histamine-mediated vascular leakage. Proc Natl Acad Sci USA 2009; 106: 14552–14557. 19622728 10.1073/pnas.0904073106 PMC2732859

[bib56] Gardai SJ, Hildeman DA, Frankel SK et al. Phosphorylation of Bax Ser184 by Akt regulates its activity and apoptosis in neutrophils. J Biol Chem 2004; 279: 21085–21095. 14766748 10.1074/jbc.M400063200

[bib57] Lee A, Whyte MK, Haslett C. Inhibition of apoptosis and prolongation of neutrophil functional longevity by inflammatory mediators. J Leukoc Biol 1993; 54: 283–288. 8409750

[bib58] Douda DN, Yip L, Khan MA, Grasemann H, Palaniyar N. Akt is essential to induce NADPH-dependent NETosis and to switch the neutrophil death to apoptosis. Blood 2014; 123: 597–600. 24458280 10.1182/blood-2013-09-526707

[bib59] Karimi K, Bienenstock J, Wang L, Forsythe P. The vagus nerve modulates CD4^+^ T cell activity. Brain Behav Immun 2010; 24: 316–323. 19887104 10.1016/j.bbi.2009.10.016

[bib60] Buijs RM, van der Vliet J, Garidou ML, Huitinga I, Escobar C. Spleen vagal denervation inhibits the production of antibodies to circulating antigens. PLoS ONE 2008; 3: e3152. 18773078 10.1371/journal.pone.0003152 PMC2519832

[bib61] Gautron L, Rutkowski JM, Burton MD, Wei W, Wan Y, Elmquist JK. Neuronal and nonneuronal cholinergic structures in the mouse gastrointestinal tract and spleen. J Comp Neurol 2013; 521: 3741–3767. 23749724 10.1002/cne.23376 PMC4081472

[bib62] Huston JM, Wang H, Ochani M et al. Splenectomy protects against sepsis lethality and reduces serum HMGB1 levels. J Immunol 2008; 181: 3535–3539. 18714026 10.4049/jimmunol.181.5.3535 PMC4533852

[bib63] Teixeira FM, Fernandes BF, Rezende AB et al. *Staphylococcus aureus* infection after splenectomy and splenic autotransplantation in BALB/c mice. Clin Exp Immunol 2008; 154: 255–263. 18782329 10.1111/j.1365-2249.2008.03728.x PMC2612725

[bib64] Chaudry IH, Tabata Y, Schleck S, Baue AE. Effect of splenectomy on reticuloendothelial function and survival following sepsis. J Trauma 1980; 20: 649–656. 7401204 10.1097/00005373-198008000-00003

[bib65] Grover GJ, Loegering DJ. Role of the liver in host defense to pneumococcus following splenectomy. J Surg Res 1984; 37: 448–452. 6513540 10.1016/0022-4804(84)90212-9

[bib66] Fernandes BF, Rezende AB, Alves CC et al. Splenic autotransplantation restores IL-17 production and antibody response to *Streptococcus pneumoniae* in splenectomized mice. Transplant Immunol 2010; 22: 195–197. 10.1016/j.trim.2009.12.002 20036332

[bib67] van Westerloo D, van der Poll T. Acute vagotomy activates the cholinergic anti-inflammatory pathway. Am J Physiol Heart Circul Physiol 2005; 288: H977–H978. 10.1152/ajpheart.00837.2004 15650159

[bib68] George MS, Sackeim HA, Rush AJ et al. Vagus nerve stimulation: a new tool for brain research and therapy. Biol Psychiatry 2000; 47: 287–295. 10686263 10.1016/s0006-3223(99)00308-x

[bib69] Matute-Bello G, Frevert CW, Kajikawa O et al. Septic shock and acute lung injury in rabbits with peritonitis: failure of the neutrophil response to localized infection. Am J Respir Crit Care Med 2001; 163: 234–243. 11208651 10.1164/ajrccm.163.1.9909034

