# Supplementary material for: Signals of vagal circuits engaging with AKT1 in α7 nAChR+CD11b+ cells lessen E. coli and LPS-induced acute inflammatory injury
Source: Cell Discov. 2017 Apr 11;3:17009–. doi: 10.1038/celldisc.2017.9 (PMC5419718; doi:10.1038/celldisc.2017.9)
Supplement: Supplementary information [file celldisc20179-s1.pdf]

Supplementary Figures

Supplementary Figure 1

Effect of vagotomy on spleen, blood and lung neutrophils, ELW, and phosphorylation of p65 NF-kB and AKT1 in splenic neutrophils in LPS-induced ALI. Sham and vagotomized mice were IT challenged with LPS (5 mg/kg). 15 h after IT LPS, mice were killed to assess (***A***) neutrophils in the spleen; (***B***) blood neutrophils; (***C***) lung MPO (an index of lung neutrophil infiltration); ELW (***D***), and p-P65 NF-kB (***E***) and p-AKT1^{ser473} levels (***F***) in splenic neutrophils. N = 4 in each group; *$P < 0.05$, *Student's* t test (***A-B, D-F***) and repeated measures two-way ANOVA was used for statistical analysis (***C***). Data are presented as mean ± SD. Representative from two independent experiments.

Supplementary Figure 2

Identification of specificity of rabbit anti-α7 nAChR polyclonal antibody. Lung, blood, and BM cells were isolated from wildtype and *Chrna7*$^{-/-}$ mice and labeled with Alexa Fluor® 488-rabbit anti-α7 nAChR antibody. The whole cell population was gated. Percentage of α7 nAChR$^+$ cells was evaluated in lung (**A-B**), blood (**C-D**), and BM (**E-F**) cells. N = 3 in each group. Data are presented as mean ± SD. Wildtype and *Chrna7*$^{-/-}$ BM cells were labeled with Fluor-633 α-bungarotoxin (Biotium) and the whole cells were gated. The α7 nAChR$^+$ cells in the BM cells were calculated (**G**).

Supplementary Figure 3

A-F. Changes of monocytes (Ly6C^{hi}Ly6G^{int}) and neutrophils (Ly6C^{int}Ly6G^{hi}) in the spleen, BM, and lung from PBS-challenged sham and vagotomized mice. Spleen, BM, and lung cells were isolated from sham + PBS (IT) and vagotomy + PBS (IT) mice at 24 h. The cells were labeled with fluorescent anti-Ly6C and Ly6G antibodies. The whole cell population was gated. Percentage of monocytes (Ly6C^{hi}Ly6G^{int}) and neutrophils (Ly6C^{int}Ly6G^{hi}) in the spleen (***A-C***), BM (***D-F***), and lung (***G-I***) were analyzed. N = 3 in each group. Data are presented as mean ± SD.

J. ***Changes of lung MPO, an index of neutrophil infiltration***. Sham and vagotomized (right cervical) wildtype mice were IT challenged with PBS and killed at 4, 12, and 24 h post PBS challenge. The lungs were excised and homogenized to measure MPO activity. N = 3 in each group. Data are presented as mean ± SD.

Supplementary Figure 4

Representative photomicrographs of lung histology in E. coli pneumonia compared sham to vagotomized mice.

(A) Effect of vagotomy on infiltration of neutrophils in E. coli infected lungs. The sham and vagotomized mice were IT challenged with *E. coli* 2.5 × 10^6 cfu and then sacrificed at 12 and 24 h. The lungs were collected for immunohistochemistry. Red color: stained with anti-VE-cadherin antibody for labeling blood vessels; green color: labeled with anti-Gr1 antibody for detecting neutrophils. A: Arterioles; B: bronchus; V: vein. Scale bar 50 µm; objective magnification × 20. ***(B) Effect of vagotomy on α7 nAChR⁺Gr1⁺ cells in E. coli infected lungs***. The lung sections were incubated with primary rabbit anti-α7 nAChR and rat-anti-Gr1 antibodies then labeled with Fluro488-anti rabbit or Fluor-594 anti rat secondary antibodies. Red color: $Gr1^+$; green color: α7 $nAChR^+$; objective magnification × 40; Scale bar 20 µm.

Supplementary Figure 5

Flow cytometry analysis of changes of α7 $nAChR^+CD11b^+$ cell population in peripheral blood from E. coli-infected sham, vagotomized and α7 nAChR-agonist supplemented vagotomized mice. Experimental setting was established in **Figure 4**. The blood cells were isolated and labeled with corresponding fluorescent antibodies. The whole cell population was divided into three gates: lymphocyte, monocyte, and PMN based on size and granularity of cells (***A, D, G***). The α7 $nAChR^+CD11b^+$ cells from each gate were subgated (***B, E, H***). The percentage of α7 $nAChR^+CD11b^+$ cells from each subgate was presented (***C, F, and I***). *P < 0.05, one-way ANOVA with *Bonferroni post hoc* test. Data are presented as mean ± SD.

Supplementary Figure 6

Activation of α7 nAChR promotes phosphorylation of p-AKT1 at Ser473 site in granulocytes and suppresses cytokine production.

A-B. The wildtype and *Chrna7*$^{-/-}$ splenic neutrophils were pretreated with α7 nAChR specific agonist-PHA568487 and then challenged them with LPS and separate cytoplasm and nucleus 1 h later. P-$AKT1^{Ser473}$ was measured in cytoplasm (***A***) and nucleus (***B***) by ELISA. ***C***. The supernatant was collected to measure CXCL2 levels by ELISA in LPS-challenged splenic neutrophils pretreated with different concentration of PHA568487. Representative of two experiments. ***D***. The splenic neutrophils were pretreated with Wortmannin for 15 min and then challenged with LPS. The

supernatant was collected to measure CXCL2 levels by ELISA 4 h later. N = 8 in each group. ***P* < 0.01. ***E***. The isolated splenic neutrophils were challenged with LPS (1 μm). The supernatant was collected to measure CXCL2 and TNF-α levels by ELISA 4 h later. N = 3 in each group. ***P* < 0.01, **P* < 0.05. Student's t test and data are presented as mean ± SD.

Supplementary Figure 7

Deficiency of AKT1 worsens BAL profiles. The wildtype and *Akt1*$^{-/-}$ mice were challenged with an IT *E. coli* (2.5×10^6 cfu). The mice were killed at 24 h after *E. coli* challenge. The BAL was collected to measure protein (***A***), *E. coli* colonies (***B***), and TNF-α level (***C***). N = 5 in each group. **P* < 0.05, Student's t test. Data are presented as mean ± SD.

Supplementary Figure 8

Effect of deletion of Chrna7 and vagotomy on BAL profiles and splenic p-AKT1, p-STAT3 and p-ERK levels in E. coli pneumonia. The wildtype, *Chrna7*$^{-/-}$, and vagotomized mice were respectively challenged with an intratracheal *E. coli* (2.5×10^6 cfu). The mice were killed at 24 h after *E. coli* challenge. The BAL was collected and spleens were harvested and homogenized. The supernatant of BAL was used to measure protein (***A***); *E. coli* cfu (***B***); TNF-α (***C***). The *p-AKT1*$^{\mathbf{Ser473}}$ (***D***), p-STAT3 (***E***) and p-ERK (***F***) levels in the supernatant of spleen homogenate were measured by ELISA. N = 4-5 in each group, **P* < 0.05, *Student's t* test. Data are presented as mean ± SD.

Supplementary Figure 9

A-D. Deletion of Chrna7 or Akt1 worsens acute lung infection and inflammation. The wildtype, *Chrna7*$^{-/-}$, or *Akt1*$^{-/-}$ mice were respectively challenged with an intratracheal *E. coli* (2.5×10^6 cfu). The mice were killed at d1 and d2 after *E. coli* challenge to measure ELW (***A***). Blood was collected to analyze (***B***) blood neutrophils; (***C***) blood monocytes at d1. N = 4-5 in each group, **P* < 0.05, ***P* < 0.01, *student's t* test. Data are presented as mean ± SD. The lungs were harvested and homogenized to measure MPO activity in the supernatant at d1 (***D***). N = 5 in each group, **P* < 0.05, Repeated Measures of *Two-way ANOVA*. Data are presented as mean ± SD.

E. Change of survival in E. coli-infected wildtype, Akt1$^{-/-}$***, and Chrna7***$^{-/-}$ ***mice.*** The wildtype, *Akt1*$^{-/-}$, and *Chrna7*$^{-/-}$ mice were IT challenged with *E. coli* (5×10^6 cfu) and followed up for 5 days. N = 10-23 in each group. Data are presented as mean ± SD. Log-rank test.

Supplementary Figure 10

A-B. Flow cytometry analysis of splenic CD4$^+$cells during E. coli pneumonia. Using the same experimental setting as **Fig. 7A-D**, the four groups of spleen cells were submitted to flow cytometric analysis. ***A***. lymphocyte population was gated. ***B***. Percentage of splenic CD4$^+$ cells. N = 4 in each group, **P* < 0.05. Data are presented as mean ± SD.

C-D. p-AKT1 and CHAT expression in the E. coli-infected Chrna7$^{-/-}$Itgam$^{-/-}$ spleens. Spleens were collected from *E. coli*-infected wildtype and *Chrna7$^{-/-}$Itgam$^{-/-}$* mice to perform western blotting at 24 h post *E. coli* IT infection. N = 3-4 in each group, **P* < 0.05. Data are presented as mean ± SD. Image J software was used for analyzing optical density.

Supplementary Figure 11

A-B. Effect of vagotomy and double deletion of Chrna7 and Itgam on lung Il22 and Cxcl2 mRNA during E. coli pneumonia. The sham, vagotomized, and *Chrna7$^{-/-}$Igtam$^{-/-}$* mice were IT challenged with *E. coli* (2.5 × 10^6 cfu). The wildtype mice receiving an IT PBS were used control. The mice were killed at 24 h after *E. coli* or PBS challenge. Lungs were excised to extract RNA for real-time PCR for *Il22 (**A**)* and *Cxcl2 (**B**)* mRNA. N = 3-4 in each group. Data are presented as mean ± SD.

C-E. Effect of vagotomy and double deletion of Chrna7 and Itgam on bone marrow monocytes and neutrophils during E. coli pneumonia. The sham, vagotomized, and *Chrna7$^{-/-}$Igtam$^{-/-}$* mice were IT challenged with *E. coli* (2.5 × 10^6 cfu). The wildtype mice receiving an IT PBS were used control. The mice were killed at 24 h after *E. coli* or PBS challenge. BM cells were isolated and labeled with fluorescent anti-Ly6C and Ly6G antibodies. ***C***. Gating strategies for monocytes and neutrophils; ***D***. Percentage of Ly6C^{hi}Ly6G^{int} monocytes; ***E***. Percentage of Ly6C^{int}Ly6G^{hi} neutrophils. N = 3-4 in each group. Data are presented as mean ± SD.

Supplementary Figure 12

TNF-α production in LPS-challenged peritoneal macrophages. Peritoneal macrophages were isolated from wildtype, *Chrna7$^{-/-}$*, *Itgam$^{-/-}$*, and *Chrna7$^{-/-}$Itgam$^{-/-}$* mice. The mice were ip injected with 3 ml of 3% (w/v) Brewer thioglycollate medium into the peritoneal cavity. Peritoneal lavage was performed 3 days post thioglycollate injection. The purified wildtype, *Chrna7$^{-/-}$*, *Itgam$^{-/-}$*, and *Chrna7$^{-/-}$Itgam$^{-/-}$* macrophages were separately challenged with LPS (1 μM) for 4 h. TNF-α in the supernatant of media was measured by ELISA. N = 3 in each group. Data are presented as mean ± SD.

Supplementary Figure 13

Vagal-α7 nAChR signals via AKT1 regulate α7 nAChR$^+$CD11b$^+$ cells by which dampen acute lung inflammatory responses. In the intact condition, vagus signals trigger release of acetylcholine in the spleen, and this event leads to phosphorylation of AKT1 in α7 nAChR$^+$CD11b$^+$ cells and confines these cells in the spleen. Disruption of vagal circuits reduces phosphorylation of AKT1 in splenic α7 nAChR$^+$CD11b$^+$ cells and facilitates spleen egress and lung recruitment of these cells. Without functional vagal circuits or α7 nAChR agonist stimulation, accumulated α7 nAChR$^+$CD11b$^+$ cells in the LPS or *E. coli* challenged lung lack of anti-bacterial propriety and propagate inflammatory responses.

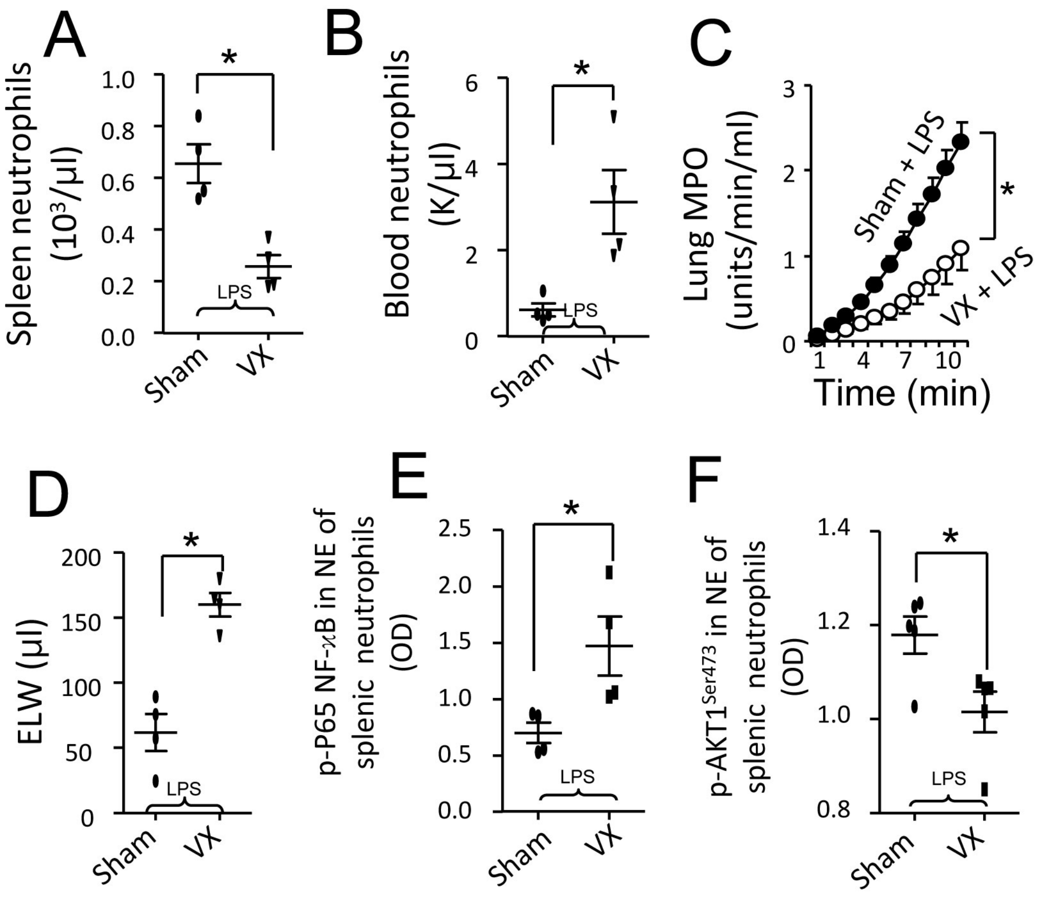

A
Spleen neutrophils (10³/µl)
1.0
0.8
0.6
0.4
0.2
0.0
*
LPS
Sham
VX
B
Blood neutrophils (K/µl)
6
4
2
0
*
LPS
Sham
VX
C
Lung MPO (units/min/ml)
3
2
1
0
Sham + LPS
VX + LPS
*
1
4
7
10
Time (min)
D
ELW (µl)
200
150
100
50
0
*
LPS
Sham
VX
E
p-P65 NF-κB in NE of splenic neutrophils (OD)
2.5
2.0
1.5
1.0
0.5
0.0
*
LPS
Sham
VX
F
p-AKT1Ser473 in NE of splenic neutrophils (OD)
1.4
1.2
1.0
0.8
*
LPS
Sham
VX

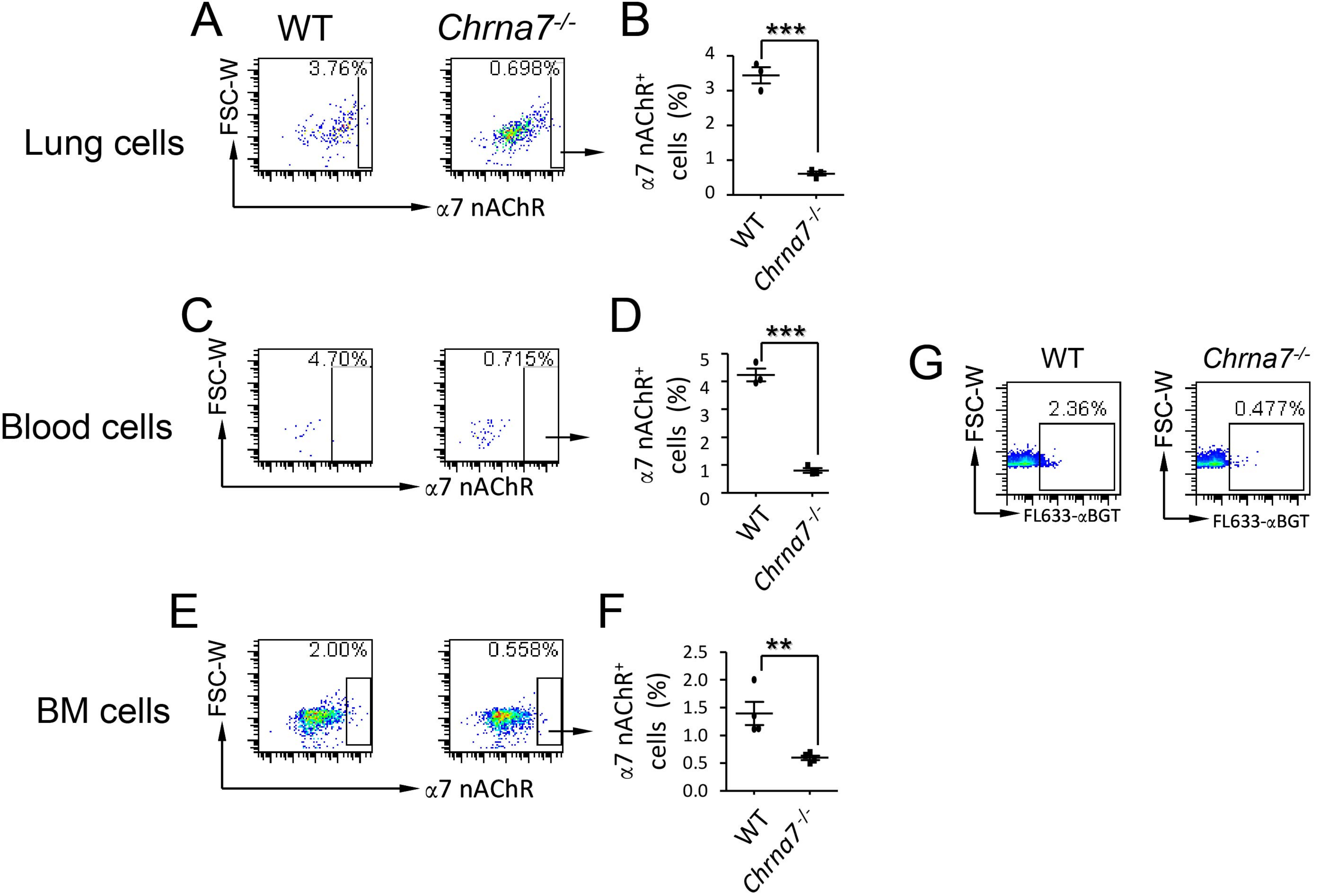

A
WT
Chrna7-/-
B
Lung cells
FSC-W
3.76%
0.698%
α7 nAChR
α7 nAChR+ cells (%)
***
WT
Chrna7-/-
C
D
Blood cells
4.70%
0.715%
G
2.36%
0.477%
FL633-αBGT
E
F
BM cells
2.00%
0.558%
**

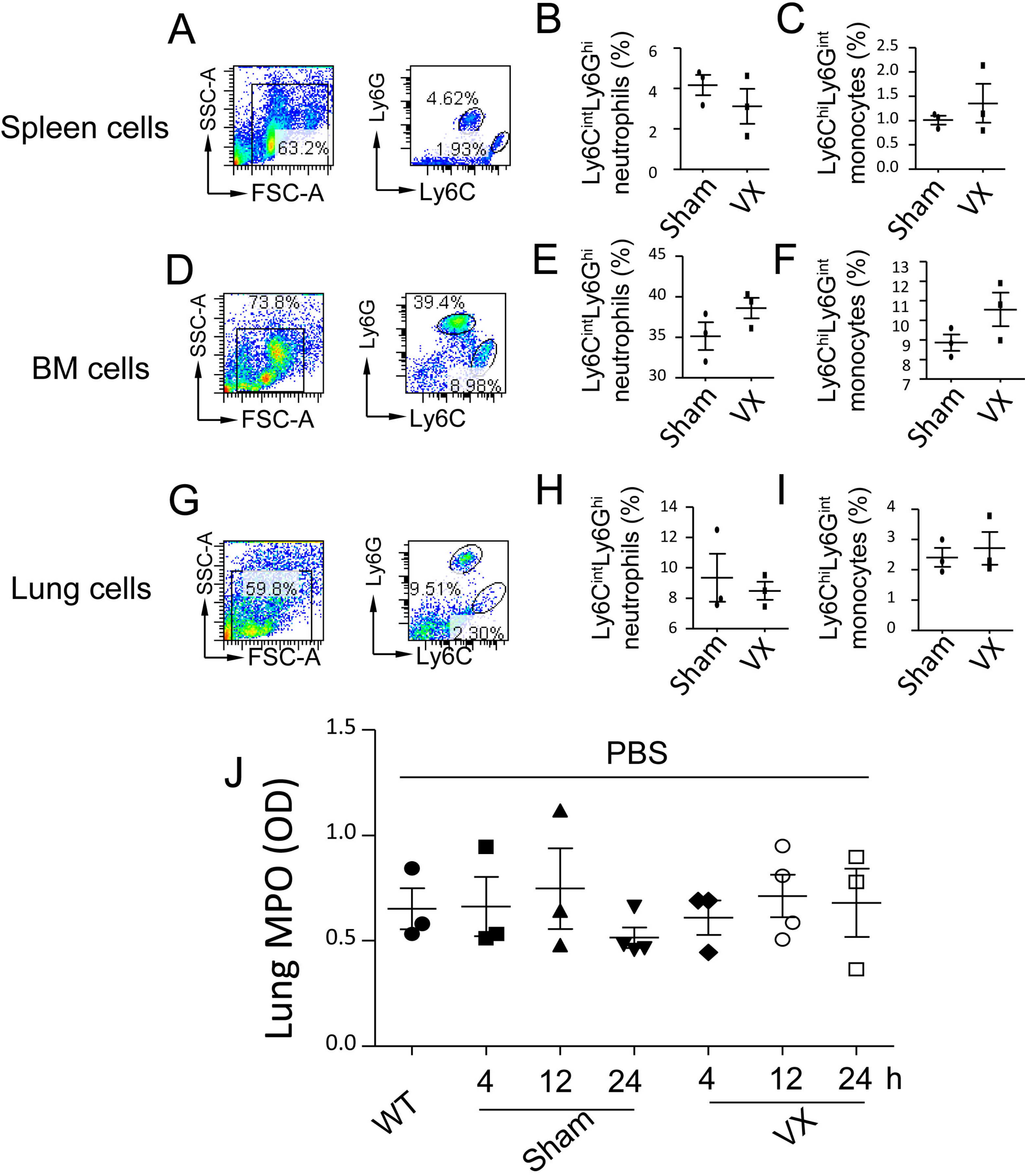
A
B
C
Spleen cells
SSC-A
FSC-A
63.2%
Ly6G
Ly6C
4.62%
1.93%
Ly6C^int Ly6G^hi neutrophils (%)
Ly6C^hi Ly6G^int monocytes (%)
Sham
VX
D
E
F
BM cells
73.8%
39.4%
8.98%
G
H
I
Lung cells
59.8%
9.51%
2.30%
J
PBS
Lung MPO (OD)
WT
4
12
24
h
Sham
VX

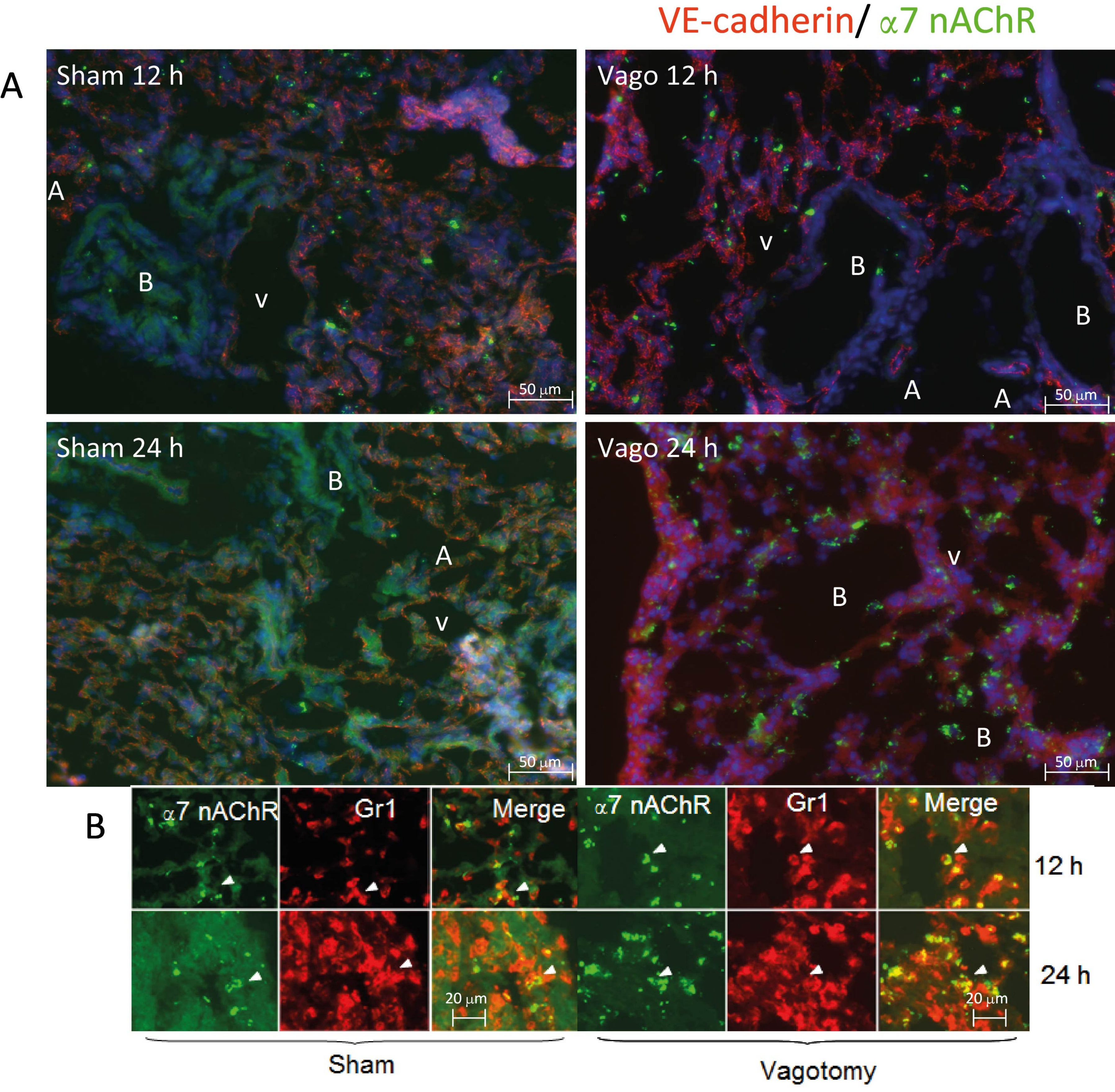
VE-cadherin/ α7 nAChR
A
Sham 12 h
A
B
v
50 μm
Vago 12 h
v
B
B
A
A
50 μm
Sham 24 h
B
A
v
50 μm
Vago 24 h
v
B
B
50 μm
B
α7 nAChR
Gr1
Merge
α7 nAChR
Gr1
Merge
12 h
24 h
20 μm
20 μm
Sham
Vagotomy

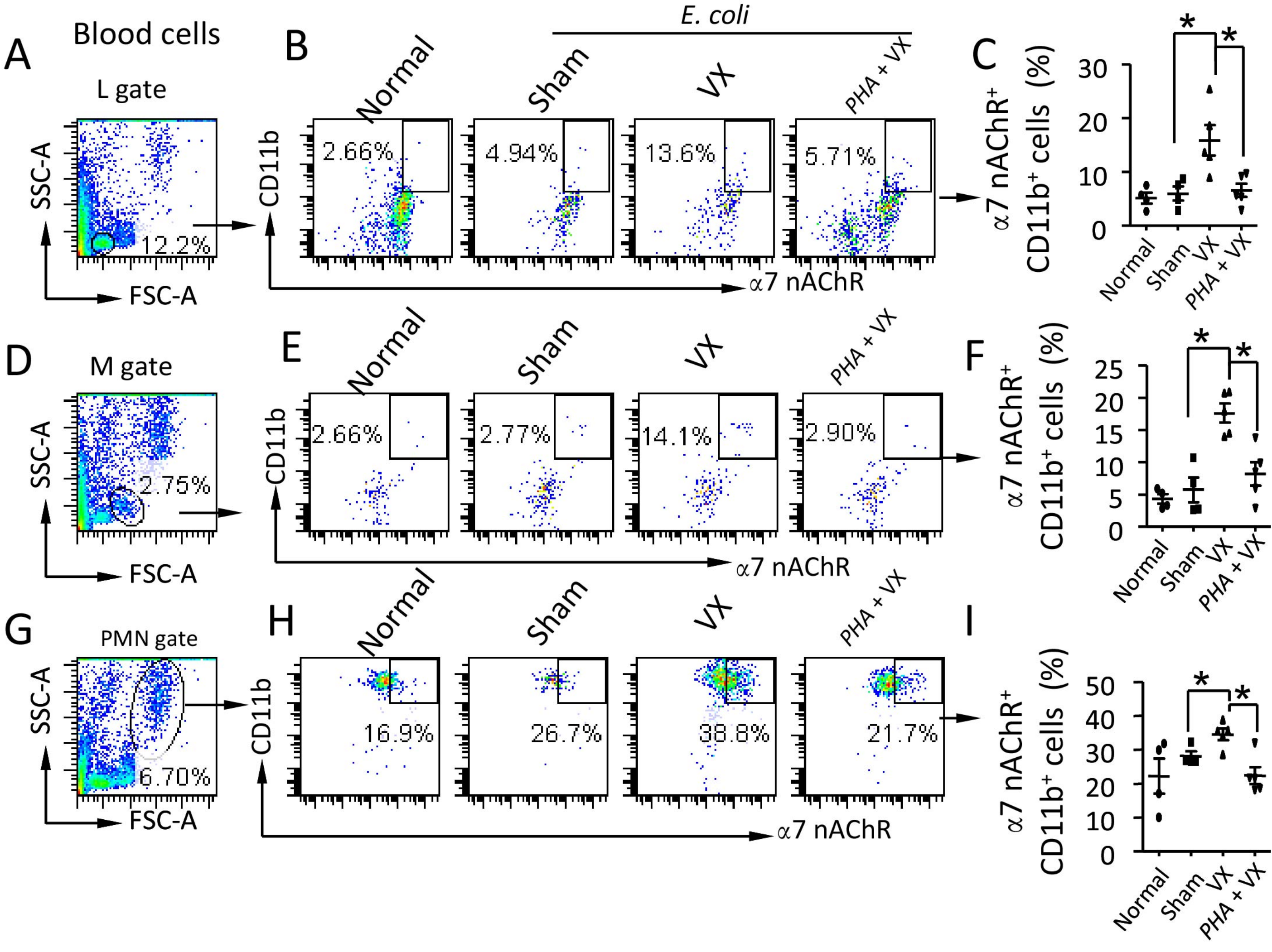

A
Blood cells
L gate
12.2%
SSC-A
FSC-A
B
E. coli
Normal
Sham
VX
PHA + VX
2.66%
4.94%
13.6%
5.71%
CD11b
α7 nAChR
C
α7 nAChR+
CD11b+ cells (%)
30
20
10
0
Normal
Sham
VX
PHA + VX
*
*
D
M gate
2.75%
SSC-A
FSC-A
E
Normal
Sham
VX
PHA + VX
2.66%
2.77%
14.1%
2.90%
CD11b
α7 nAChR
F
α7 nAChR+
CD11b+ cells (%)
25
20
15
10
5
0
Normal
Sham
VX
PHA + VX
*
*
G
PMN gate
6.70%
SSC-A
FSC-A
H
Normal
Sham
VX
PHA + VX
16.9%
26.7%
38.8%
21.7%
CD11b
α7 nAChR
I
α7 nAChR+
CD11b+ cells (%)
50
40
30
20
10
0
Normal
Sham
VX
PHA + VX
*
*

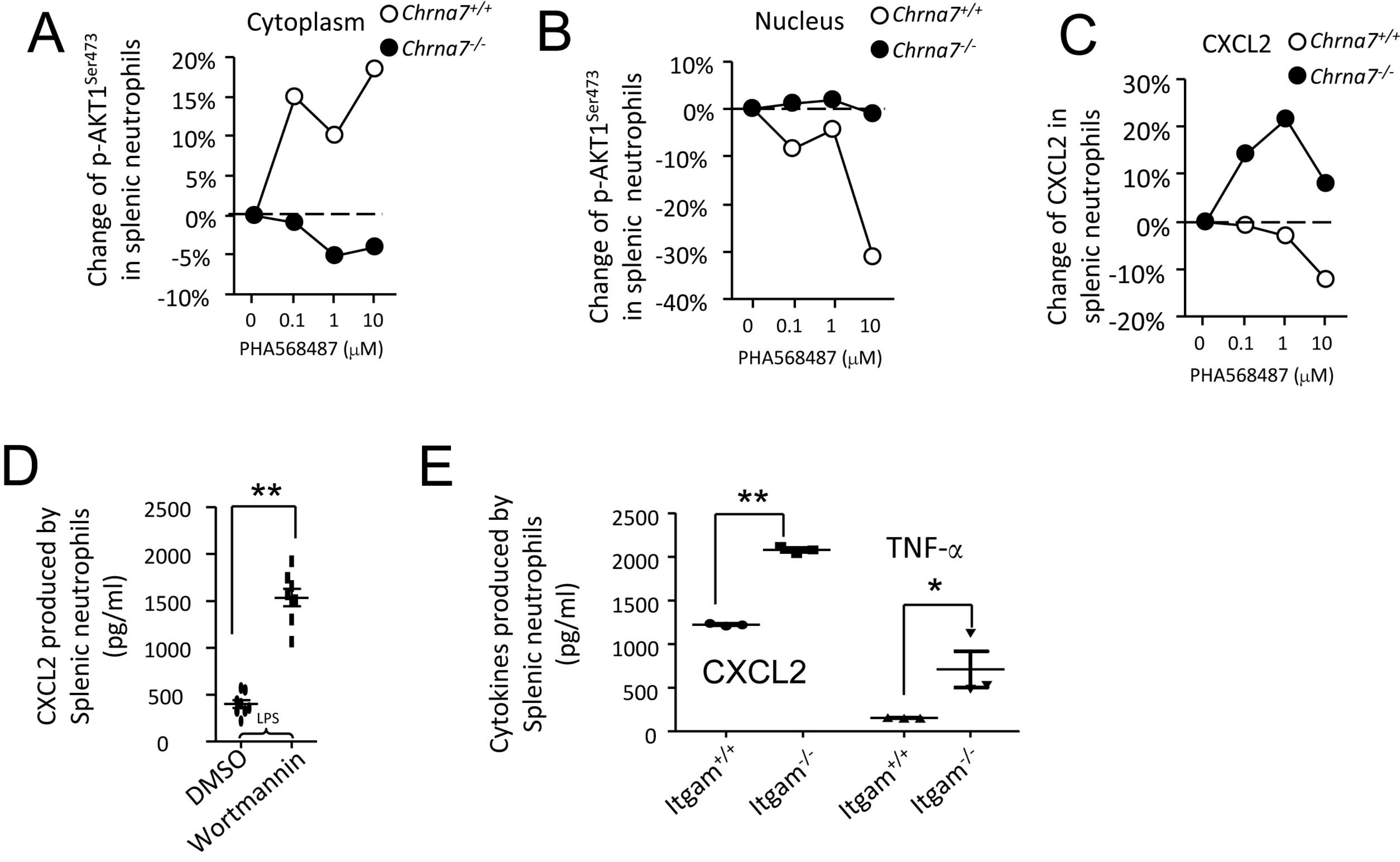
A
Cytoplasm
Chrna7+/+
Chrna7-/-
Change of p-AKT1Ser473 in splenic neutrophils
20%
15%
10%
5%
0%
-5%
-10%
0
0.1
1
10
PHA568487 (μM)
B
Nucleus
Chrna7+/+
Chrna7-/-
Change of p-AKT1Ser473 in splenic neutrophils
10%
0%
-10%
-20%
-30%
-40%
0
0.1
1
10
PHA568487 (μM)
C
CXCL2
Chrna7+/+
Chrna7-/-
Change of CXCL2 in splenic neutrophils
30%
20%
10%
0%
-10%
-20%
0
0.1
1
10
PHA568487 (μM)
D
**
CXCL2 produced by Splenic neutrophils (pg/ml)
2500
2000
1500
1000
500
0
LPS
DMSO
Wortmannin
E
**
TNF-α
*
Cytokines produced by Splenic neutrophils (pg/ml)
2500
2000
1500
1000
500
0
CXCL2
Itgam+/+
Itgam-/-
Itgam+/+
Itgam-/-

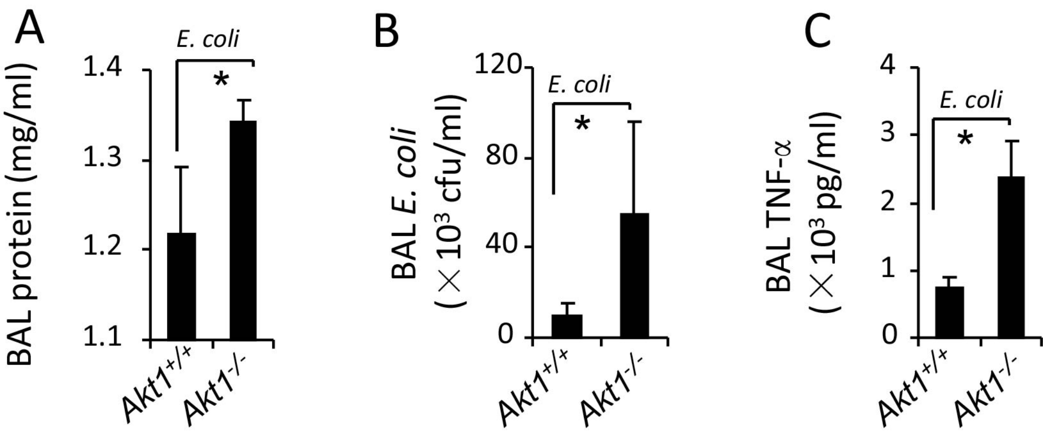

A
E. coli
*
BAL protein (mg/ml)
1.4
1.3
1.2
1.1
Akt1+/+
Akt1-/-
B
120
80
40
0
E. coli
*
BAL E. coli (×10³ cfu/ml)
Akt1+/+
Akt1-/-
C
4
3
2
1
0
E. coli
*
BAL TNF-α (×10³ pg/ml)
Akt1+/+
Akt1-/-

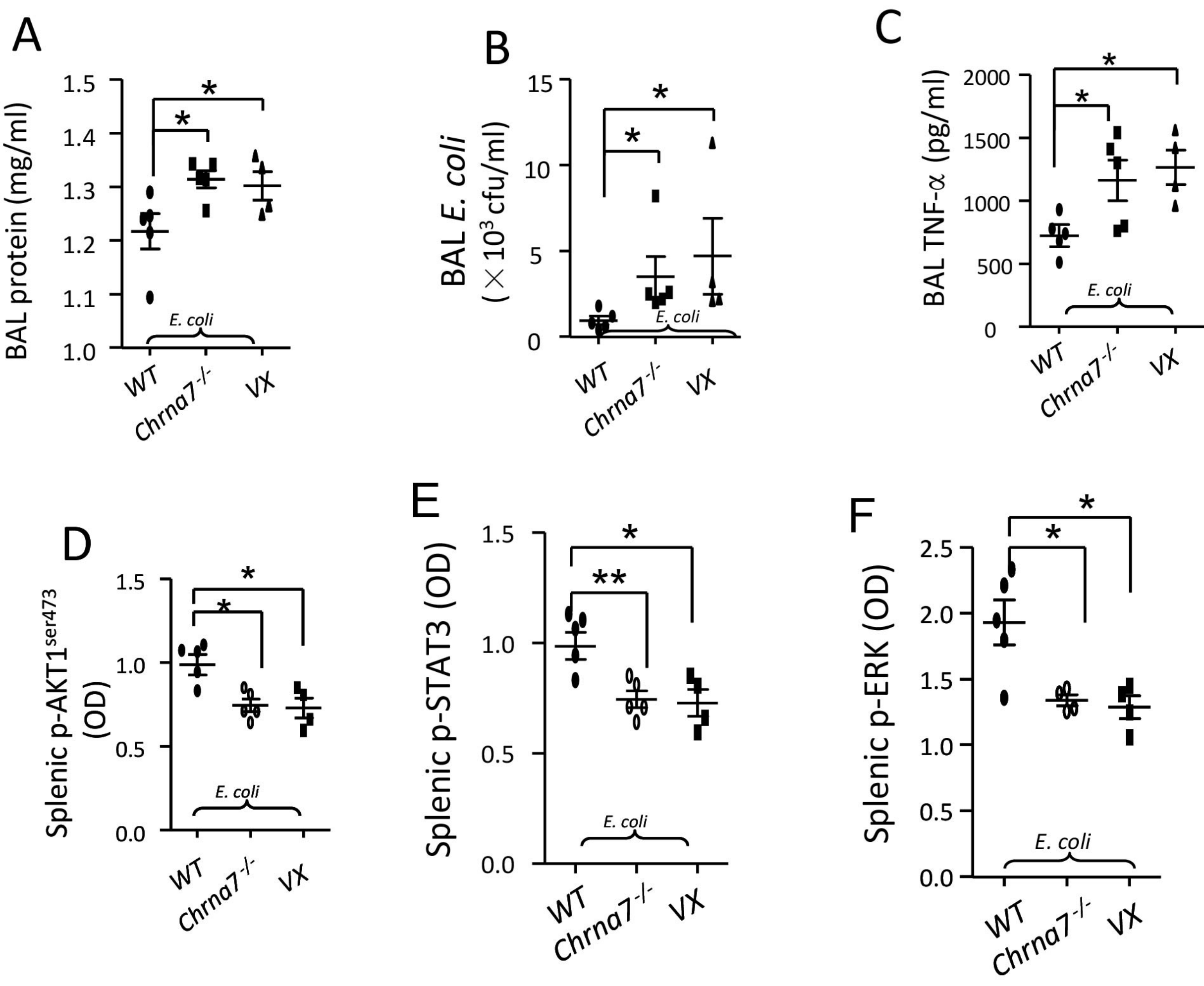

A
BAL protein (mg/ml)
1.0
1.1
1.2
1.3
1.4
1.5
E. coli
WT
Chrna7-/-
VX
B
BAL E. coli (×10^3 cfu/ml)
0
5
10
15
C
BAL TNF-α (pg/ml)
0
500
1000
1500
2000
D
Splenic p-AKT1ser473 (OD)
0.0
0.5
1.0
1.5
E
Splenic p-STAT3 (OD)
F
Splenic p-ERK (OD)
2.0
2.5
*
**

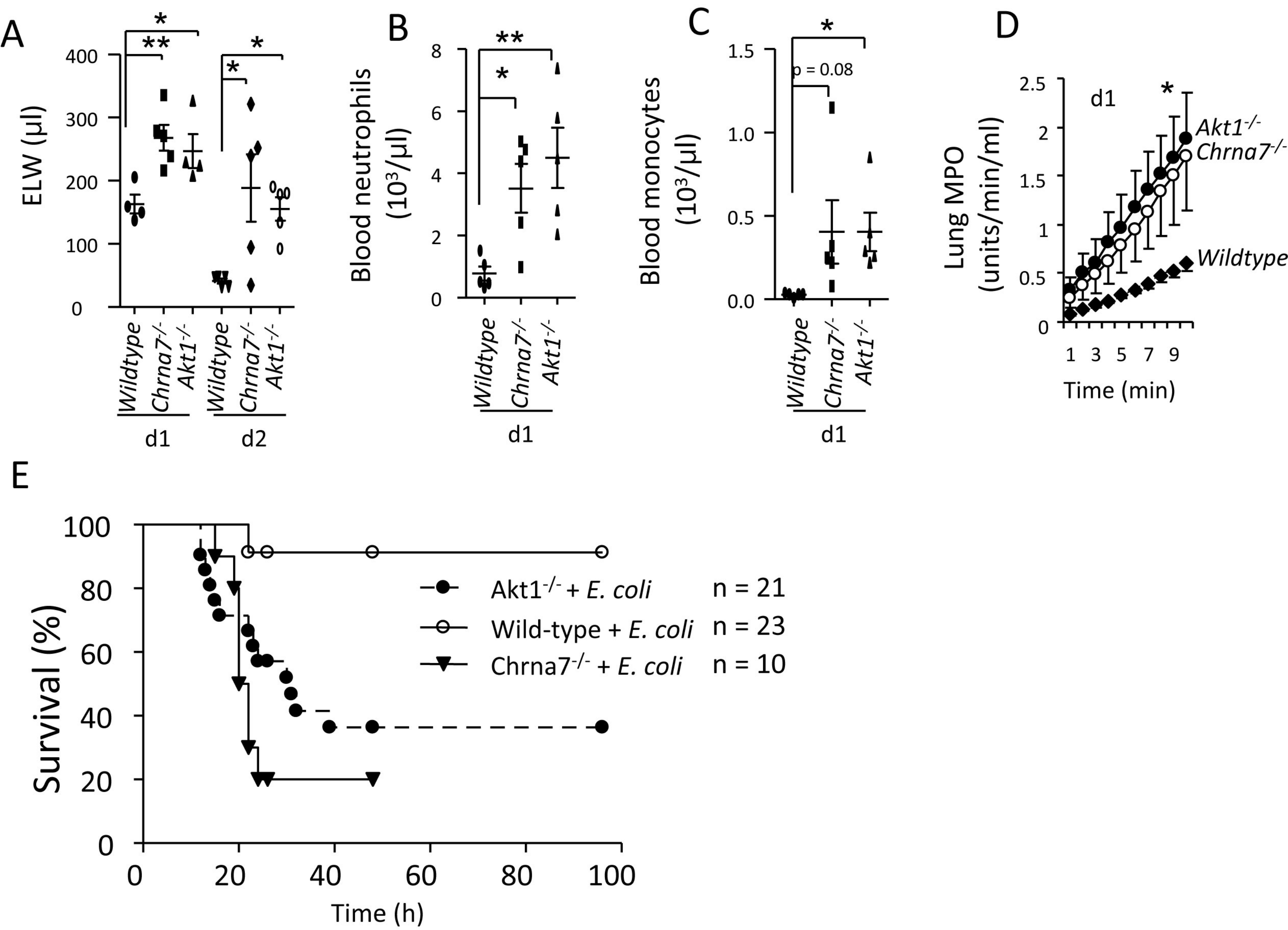

A
ELW (µl)
400
300
200
100
0
*
**
*
*
Wildtype
Chrna7-/-
Akt1-/-
Wildtype
Chrna7-/-
Akt1-/-
d1
d2
B
Blood neutrophils (10³/µl)
8
6
4
2
0
**
*
Wildtype
Chrna7-/-
Akt1-/-
d1
C
Blood monocytes (10³/µl)
1.5
1.0
0.5
0.0
*
p = 0.08
Wildtype
Chrna7-/-
Akt1-/-
d1
D
Lung MPO (units/min/ml)
2.5
2
1.5
1
0.5
0
d1
*
Akt1-/-
Chrna7-/-
Wildtype
1
3
5
7
9
Time (min)
E
Survival (%)
100
80
60
40
20
0
Akt1-/- + E. coli n = 21
Wild-type + E. coli n = 23
Chrna7-/- + E. coli n = 10
0
20
40
60
80
100
Time (h)

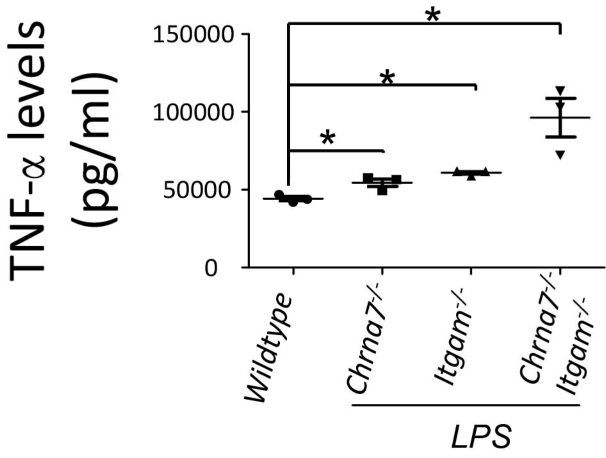
TNF-α levels (pg/ml)
150000
100000
50000
0
*
*
*
Wildtype
Chrna7-/-
Itgam-/-
Chrna7-/- Itgam-/-
LPS

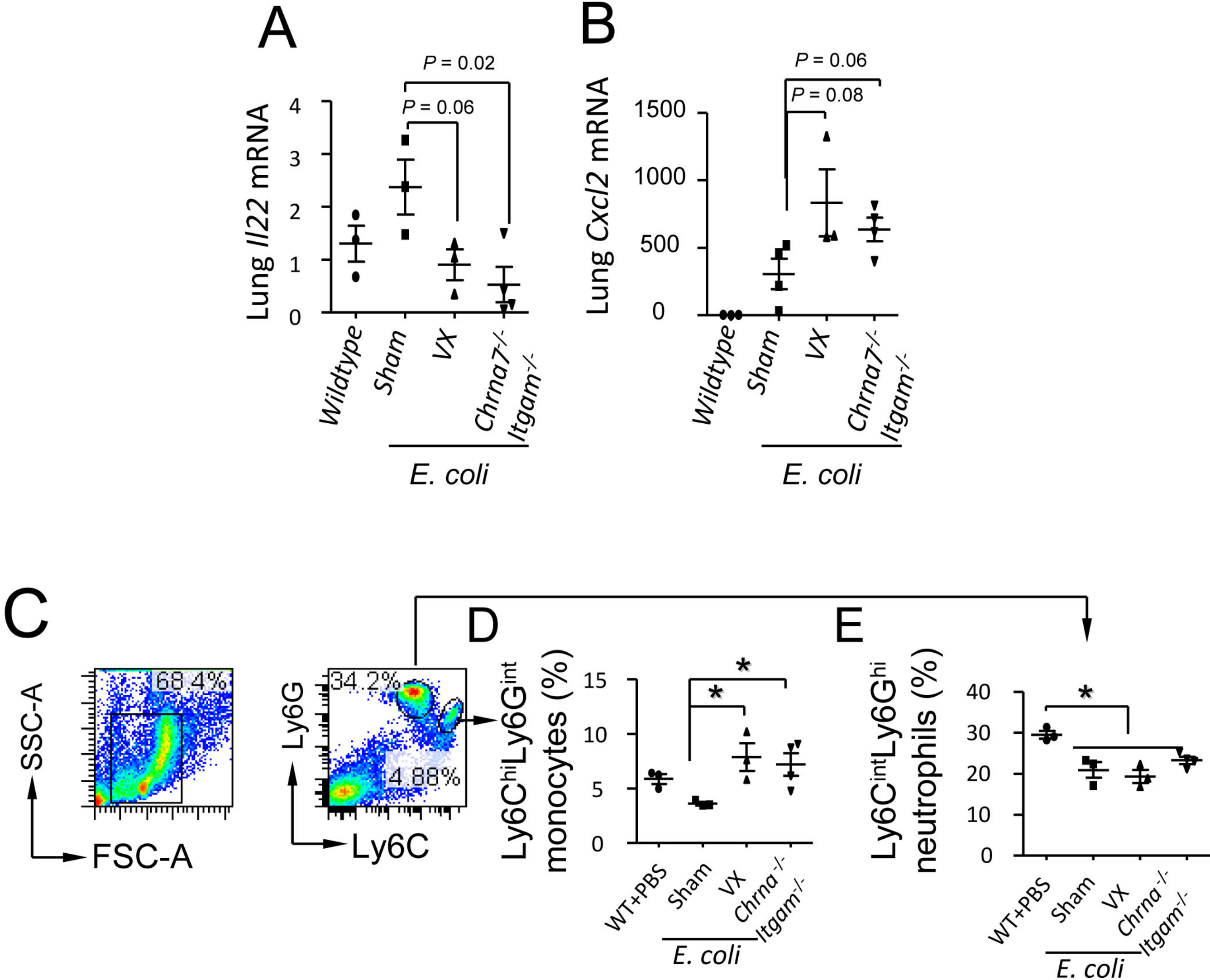
A
B
P = 0.02
P = 0.06
Lung Il22 mRNA
4
3
2
1
0
Wildtype
Sham
VX
Chrna7-/-
Itgam-/-
E. coli
P = 0.06
P = 0.08
Lung Cxcl2 mRNA
1500
1000
500
0
C
SSC-A
FSC-A
68.4%
Ly6G
Ly6C
34.2%
4.88%
D
Ly6ChiLy6Gint
monocytes (%)
15
10
5
0
*
WT+PBS
Chrna-/-
E
Ly6CintLy6Ghi
neutrophils (%)
40
30
20
10

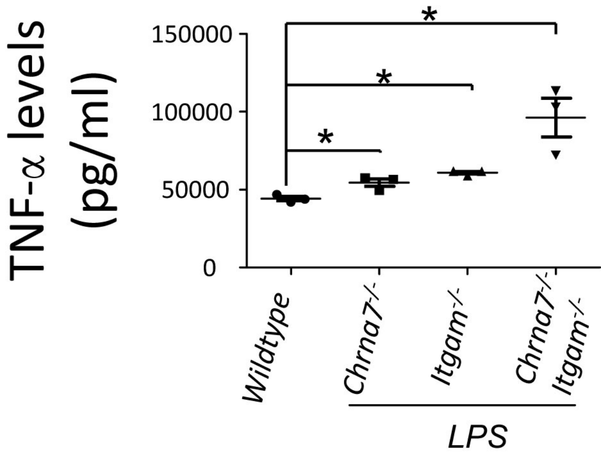

TNF-α levels (pg/ml)
150000
100000
50000
0
*
*
*
Wildtype
Chrna7-/-
Itgam-/-
Chrna7-/- Itgam-/-
LPS

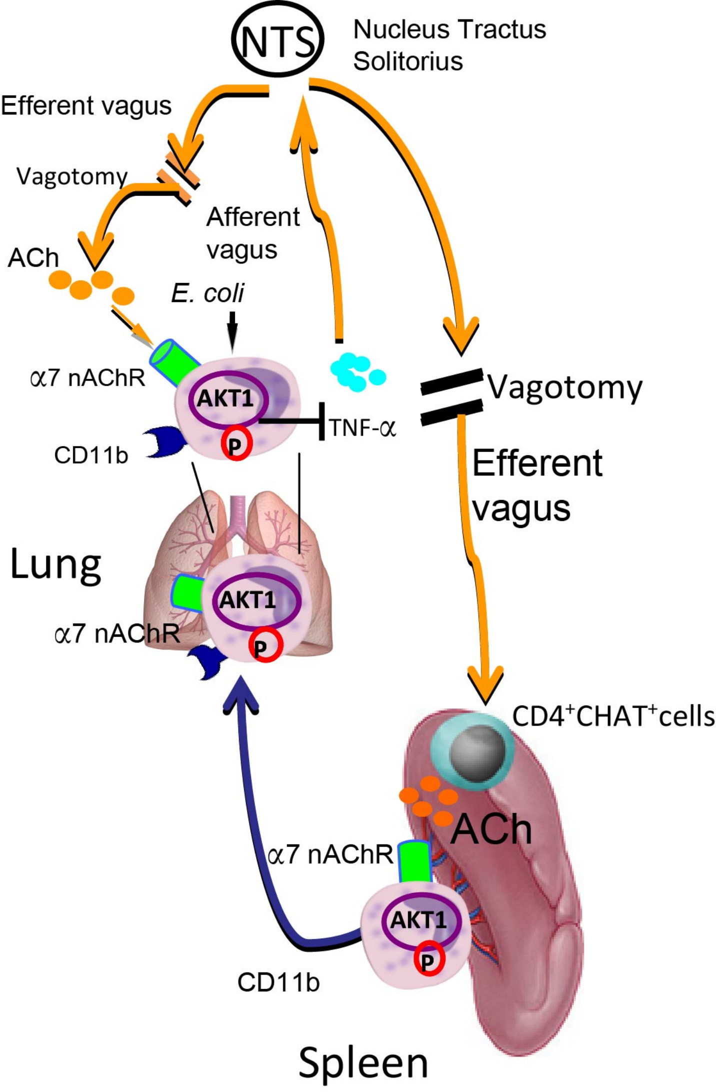

NTS
Nucleus Tractus Solitorius
Efferent vagus
Vagotomy
Afferent vagus
ACh
E. coli
α7 nAChR
AKT1
P
TNF-α
CD11b
Vagotomy
Efferent vagus
Lung
AKT1
P
α7 nAChR
CD4+CHAT+cells
ACh
α7 nAChR
AKT1
P
CD11b
Spleen
